# A Preliminary Novel CBCT-Based Staging System for Temporo-Mandibular Joint Disorders—A Systematic Review

**DOI:** 10.3390/medicina62102031

**Published:** 2026-10-20

**Authors:** Ana-Maria Cristina Arcaș, Rosana Mihaela Manea, Vasile Cǎlin Arcaș, Adriana Saceleanu, Laura Ştef, Sorin Radu Fleacǎ, Anca Maria Frǎțilǎ

**Affiliations:** 1 County Emergency Clinical Hospital Brasov, 500326 Brasov, Romania; arcascristina@yahoo.com; 2 Faculty of Medicine, Transilvania University of Brașov, 500019 Brasov, Romania; 3 Faculty of Medicine, Lucian Blaga University of Sibiu, 550169 Sibiu, Romania; laura.stef@ulbsibiu.ro (L.Ş.); radu.fleaca@ulbsibiu.ro (S.R.F.); anca.fratila@ulbsibiu.ro (A.M.F.); 4 Military Clinical Emergency Hospital of Sibiu, 550024 Sibiu, Romania; 5 Sibiu County Emergency Clinical Hospital, 550245 Sibiu, Romania

**Keywords:** temporomandibular joint disorders, temporomandibular joint, cone-beam computed tomography, osteoarthritis, diagnostic imaging, disease progression, classification, mandibular condyle

## Abstract

*Background and Objectives*: Temporomandibular disorders (TMDs) are heterogeneous conditions that may involve structural alterations of the temporomandibular joint (TMJ). Although cone-beam computed tomography (CBCT) enables detailed assessment of osseous structures, existing classifications mainly address clinical diagnosis, internal derangement, or isolated imaging findings. This review aimed to identify recurrent TMJ osseous changes and develop a practical CBCT-based staging system. *Materials and Methods*: The review was conducted according to PRISMA 2020. PubMed, Embase, and the Cochrane Library were systematically searched, supplemented by citation and grey-literature searches. Methodological quality was assessed using the Newcastle–Ottawa Scale, Joanna Briggs Institute tools, and AMSTAR 2, while evidence certainty was evaluated using GRADE. Recurrent imaging findings, clinical correlations, prognostic implications, and therapeutic perspectives were synthesized to develop the proposed scale. *Results*: Sixty-seven peer-reviewed studies were included, while 12 grey-literature records were retained as complementary evidence. Frequently reported findings included condylar flattening, erosion, sclerosis, osteophytes, cortical alterations, subchondral cystic changes, joint-space abnormalities, remodeling, dimensional reduction, and deformation. Imaging-clinical correlations were heterogeneous. The synthesis supported a four-stage system ranging from preserved osseous morphology (Stage I), through early positional/adaptive changes (Stage II) and established remodeling (Stage III), to advanced structural degeneration with marked deformation and joint-space collapse (Stage IV). *Conclusions*: CBCT-detectable TMJ osseous changes can be organized into a four-stage severity framework intended to standardize structural assessment. The scale should complement, rather than replace, clinical diagnosis or MRI evaluation and requires external and longitudinal validation before routine clinical use.

## 1. Introduction

Temporomandibular joint disorders (TMDs) are a heterogeneous group of conditions affecting the temporomandibular joint (TMJ), masticatory muscles and associated craniofacial structures [1]. They can present through a broad spectrum of clinical manifestations, ranging from mild orofacial pain to severe functional impairment [2,3,4]. In this context, standardized diagnostic frameworks are essential for both clinical practice and research [5].

The Research Diagnostic Criteria for Temporomandibular Disorders (RDC/TMD) [5] and, later, the Diagnostic Criteria for Temporomandibular Disorders (DC/TMD) were developed to standardize the diagnosis of TMDs [6]. These systems are valuable for practitioners, as they offer a classification of TMDs into well-defined categories by integrating physical examination findings with psychosocial assessment [5,6,7]. However, they remain primarily clinical diagnostic tools and, therefore, their ability to provide a thorough characterisation of morphologic joint damage is limited [8]. For this purpose, an imaging-based classification may be better suited [9,10].

The Wilkes classification is one of the most frequently cited imaging-based systems for TMD assessment, providing a structured framework for disease stratification. Similarly, the Piper classification incorporates magnetic resonance imaging (MRI) findings into the evaluation of TMJ disorders. More recent MRI-based classifications have also attempted to correlate internal derangement with clinical manifestations, further emphasizing the role of imaging in TMD assessment [11].

MRI is widely regarded as the reference imaging modality for evaluating TMJ soft-tissue structures, particularly the articular disc and associated abnormalities. However, its availability in routine dental radiology settings may be limited by higher costs, longer examination times, and the need for referral to specialized imaging centers [12].

Other investigations, such as arthrography and arthroscopy, may provide additional diagnostic information but are less commonly performed in routine clinical practice [13,14]. Moreover, some existing classifications are complex, difficult to apply consistently, or provide limited information regarding osseous alterations [11].

On the other end of the spectrum, Cone Beam Computed Tomography (CBCT) is widely available in dental radiology settings and allows high-resolution evaluation of the osseous components of the temporomandibular joint [14]. Most CBCT-based studies regarding TMDs describe individual radiologic findings without integrating these parameters into a practical and reproducible staging system [15,16]. Consequently, we identified an important gap in the literature with respect to a validated CBCT-based classification system, capable of grading TMJ osseous structural severity and of correlating radiologic stages with clinical manifestations.

Importantly, not all osseous alterations identified on CBCT necessarily indicate pathological degeneration. Therefore, these findings should be interpreted in conjunction with other structural abnormalities and the clinical context, rather than being considered independent indicators of disease severity.

Accordingly, this systematic review aims to identify and synthesize the principal CBCT-detectable osseous alterations associated with temporomandibular joint disorders and to propose a preliminary four-stage conceptual framework based on their hypothesized structural severity, intended for subsequent methodological and clinical validation.

## 2. Materials and Methods

This study was designed as a systematic literature review, conducted and reported in accordance with the PRISMA 2020 guidelines [17], with the additional objective of developing a preliminary, evidence-informed CBCT-based structural severity framework based on the synthesized findings.

### 2.1. Literature Search Strategy

To ensure a comprehensive review, an extensive literature search was conducted across multiple electronic databases, which included PubMed, Embase and the Cochrane Library. The search strategy was executed by three reviewers. Each reviewer was assigned one database and independently performed the search according to a predefined strategy. After completion, all identified records were combined into a single database for further screening. First, duplicate records were identified and removed. The remaining studies were then screened by title, followed by abstract screening. Studies considered potentially eligible were then evaluated in full text according to the predefined inclusion and exclusion criteria. Disagreements regarding eligibility at any stage of the selection process were resolved through discussion, majority decision and final consensus among the researchers.

Each researcher was responsible for searching and initially screening records from an assigned database. Following duplicate removal, potentially eligible reports underwent full-text assessment according to predefined eligibility criteria. Uncertain eligibility decisions were discussed among the three researchers and resolved by consensus. However, independent duplicate screening of all records was not undertaken. This methodological limitation was considered when interpreting the findings, as the absence of systematic duplicate screening may have increased the risk of selection errors.

Studies were included if they met the following criteria: articles published in English between January 2021 and March 2026; studies involving patients with TMDs with visible radiologic changes; studies evaluating osseous changes of the temporomandibular joint using CBCT, CT, MRI or other imaging methods; and studies describing diagnostic, prognostic, therapeutic or classification-related aspects of TMD. Studies were excluded if they were not written in English, were not published within the predefined time frame or did not focus on TMD or TMJ pathology. Articles were also excluded if they addressed only muscular changes in TMD without joint involvement, did not include imaging assessment, did not report osseous or structural TMJ changes, or focused exclusively on unrelated craniofacial conditions. Case reports, editorials, letters to the editor, conference abstracts without full text, animal studies, and studies with insufficient methodological information were also excluded.

MeSH terms and free-text keywords were combined using Boolean operators (Table 1) [18,19] The PubMed strategy combined MeSH terms with title/abstract keywords, the Embase strategy used Emtree terms and title/abstract fields, while the Cochrane Library strategy used broader free-text terms adapted to its search interface.

Additionally, a second structured search was performed in the same databases in order to identify studies addressing existing classification systems for TMDs (Table 2).

The primary search strategy was designed to identify studies addressing CBCT-detectable osseous alterations of the temporomandibular joint, particularly those investigating their clinical relevance. Consequently, the search incorporated terms related to clinical manifestations and imaging findings. Although this approach increased the specificity of the search, it may have excluded studies describing osseous morphology without explicit reference to clinical symptoms. Supplementary citation searching was therefore used to identify additional relevant publications. Studies employing CT or MRI were considered eligible when they provided relevant structural information, despite the primary search being focused on CBCT.

### 2.2. Grey Literature Search

In addition to the database search described above, a grey literature search was performed, using Google Scholar, institutional repositories, thesis databases, and relevant professional or academic sources. Search terms included combinations of keywords such as: temporomandibular disorders, temporomandibular joint, CBCT, cone-beam computed tomography, osseous changes, degenerative joint disease, osteoarthritis, condylar remodeling, erosion, sclerosis, osteophytes, joint space, classification, staging, and clinical manifestations.

The grey literature search was conducted during the same predefined publication timeframe (January 2021–March 2026). The search and screening procedures were documented separately from the primary database search. Additional details regarding the sources consulted, search strategies, retrieved records, and eligibility decisions are available in the online repository.

Potentially relevant records were screened by title and available summary or abstract, and full texts were assessed when accessible.

### 2.3. Risk of Bias Assessment

The methodological quality and risk of bias of the included studies were evaluated using design-specific appraisal tools. The Newcastle–Ottawa Scale (NOS) was applied to observational cohort, case–control, and comparable non-randomized studies [20]; Joanna Briggs Institute (JBI) critical appraisal tools were used for analytical cross-sectional, quasi-experimental, diagnostic accuracy, and randomized clinical studies [21]; and AMSTAR 2—A Measurement Tool to Assess Systematic Reviews 2, was applied to systematic reviews and evidence syntheses [21].

For NOS-assessed studies, three domains were evaluated: selection, comparability, and outcome/exposure assessment. These included representativeness of the study population, appropriateness of comparison groups, definition of exposure or outcomes, control of relevant confounders, objectivity of outcome assessment, adequacy of follow-up, and appropriateness of statistical analysis. Particular attention was paid to CBCT/MRI methodology, definition of osseous findings, examiner calibration, and inter- or intra-observer reliability [20].

JBI tools were selected according to study design. The 8-item checklist for analytical cross-sectional studies assessed inclusion criteria, population and setting, exposure and outcome measurement, confounding, and statistical analysis. The 9-item quasi-experimental checklist evaluated group comparability, presence of comparison/control groups, repeated measurements, follow-up, outcome reliability, and statistical methods. The 10-item diagnostic accuracy checklist assessed patient selection, index-test and reference-standard validity, independent interpretation, timing between tests, completeness of verification, and analysis. The 13-item randomized-trial checklist evaluated randomization, allocation concealment, baseline comparability, blinding, treatment consistency, follow-up, intention-to-treat analysis, outcome measurement, statistical analysis, and trial design. Because JBI tools differ by study type, scores with different denominators were not considered directly interchangeable [22].

AMSTAR 2 assessed systematic reviews regarding protocol development, eligibility criteria, justification of study designs, search comprehensiveness, duplicate study selection and data extraction, reporting of excluded studies, description of included studies, risk-of-bias assessment, funding, meta-analytical methods, consideration of bias and heterogeneity, publication bias, and conflicts of interest [21].

All 16 AMSTAR 2 items were evaluated for each included systematic review. However, the overall methodological confidence assessment was based on the seven predefined critical domains (items 2, 4, 7, 9, 11, 13, and 15). The complete 16-item assessments are available in the OSF repository.

All assessments were performed independently using standardized forms. Disagreements were resolved through discussion, majority decision, and final consensus. Non-applicable criteria were recorded as such and did not reduce the final methodological rating.

### 2.4. Data Extraction and Strength of Evidence

Following study selection, each researcher extracted the data relevant to the objectives of the present article using standardized extraction forms. For the first search, focused on CBCT-detectable osseous TMJ changes, extracted data included: author, year, study design, population, imaging method, diagnostic criteria, osseous findings, clinical manifestations, imaging-clinical correlations, treatment implications, and limitations. For the second search, focused on existing TMD/TMJ classification systems, extracted data included: author, year, classification name, diagnostic basis, imaging method, staging principles, number of stages or categories, clinical applicability, limitations, and relevance for comparison with the proposed CBCT-based classification.

Information extracted from eligible grey literature sources was used only as complementary contextual evidence and was not considered equivalent to peer-reviewed evidence.

Data extraction was initially performed by the researcher responsible for each assigned database using standardized extraction forms. Subsequently, the extracted information from the included studies was independently verified by a second researcher to identify potential inaccuracies or omissions. Any discrepancies were discussed and resolved by consensus among the research team.

The quality of evidence derived from the literature review was assessed using the Grading of Recommendations Assessment, Development and Evaluation (GRADE) approach for the main radiologic-clinical associations and therapeutic implications identified across the included studies [23]. The certainty of evidence was classified as high, moderate, low, or very low for the main outcomes related to CBCT-detectable osseous TMJ changes, clinical correlation, and treatment relevance [23,24].

The GRADE framework was adapted for a narrative assessment of evidence certainty, considering methodological limitations, consistency, indirectness, imprecision, and potential publication bias. Given the descriptive nature and heterogeneity of the included studies, certainty judgments were applied to the main findings rather than to pooled outcome estimates. These assessments were intended to contextualize the proposed framework rather than establish its validity.

### 2.5. Development of the Proposed CBCT-Based Scale

The proposed CBCT-based classification was developed through a narrative synthesis of recurrent osseous alterations identified in the systematic review. These findings were grouped according to their morphological characteristics and hypothesized structural severity, forming the basis of a preliminary four-stage framework. The classification represents an evidence-informed conceptual proposal rather than a formally validated instrument. Content validity, standardized staging criteria, and reproducibility will be evaluated in subsequent research.

The four-stage structure was selected as a pragmatic conceptual organization of the radiological findings identified in the literature, ranging from preserved osseous morphology to increasingly extensive structural alterations. The assignment of individual findings to each stage was based on their hypothesized morphological severity rather than on validated quantitative thresholds or demonstrated chronological progression. No formal Delphi consensus process, content-validity assessment, or pilot reliability testing was performed. Consequently, the proposed categories should be regarded as descriptive rather than operational diagnostic criteria, and cases exhibiting overlapping features cannot yet be assigned to a stage using a validated decision algorithm.

### 2.6. Protocol Registration and Ethical Considerations

The review protocol was registered on the Open Science Framework under the registration code osf.io/z3bt5 [25] on 9 May 2026. Registration occurred before the literature search and screening process. No subsequent amendments were made to the registered protocol. The review was reported in accordance with PRISMA 2020 guidelines.

The repository contains the raw extracted data, PRISMA checklist (Supplementary File S1), search strategies, study characteristics, risk-of-bias assessments, excluded-study records, and additional graphical materials. Further information is available from the corresponding author upon reasonable request.

## 3. Results

### 3.1. Database Search Results

The structured database search identified a total of 501 records, including 13 records from PubMed, 462 from Embase, and 26 from the Cochrane Library. Before screening, 42 records were removed, consisting of 11 duplicates and 31 narrative reviews or overview articles that did not meet the predefined eligibility criteria. After this step, 459 records remained for title and abstract screening.

During title and abstract screening, 362 records were excluded because they were not directly related to temporomandibular joint osseous alterations, did not include relevant imaging assessment, focused on unrelated craniofacial or musculoskeletal conditions, or did not provide information applicable to the development of a CBCT-based classification system. Consequently, 97 reports were sought for retrieval.

**Figure 1:**
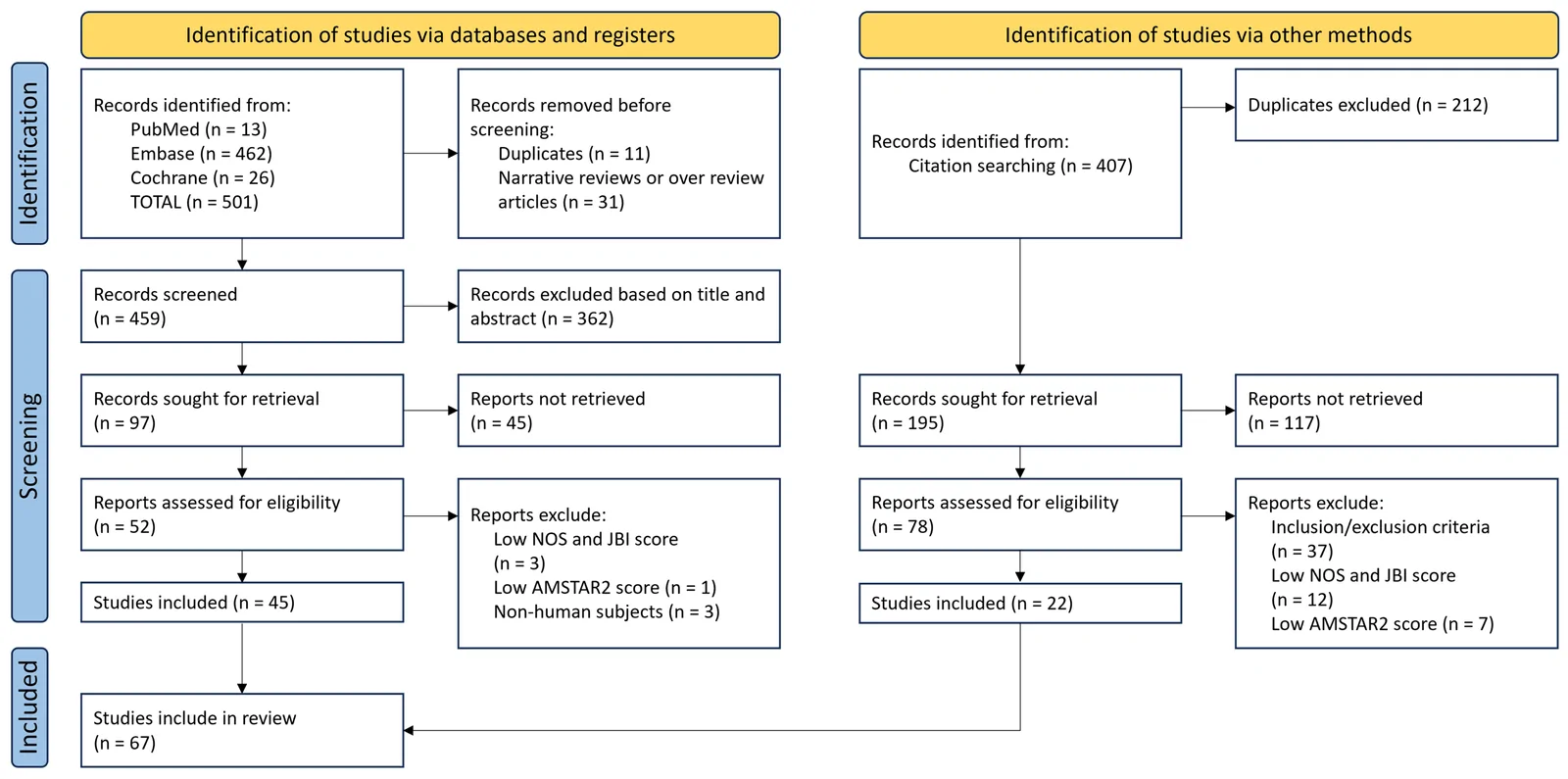
PRISMA 2020 flow diagram showing the identification, screening, eligibility assessment, and inclusion of studies [18].

Of these, 45 reports could not be retrieved, leaving 52 full-text reports for eligibility assessment. The inability to retrieve these reports was documented during the study selection process. As their full texts were unavailable, their eligibility could not be definitively assessed. After full-text evaluation, 45 studies identified through database searching were included in the review. The remaining seven full-text reports were excluded following eligibility assessment, with the specific reasons for exclusion documented in the supplementary excluded-study records available in the OSF repository. The complete database selection process is presented in the PRISMA 2020 flow diagram in Figure 1.

### 3.2. Other Sources Search Results

In addition to the database search, supplementary citation searching identified 407 records. After duplicate removal, 195 reports remained and were sought for retrieval.

Of these, 117 reports could not be retrieved as full-text documents. After full-text evaluation of the remaining 78 reports, 37 were excluded because they did not meet the predefined eligibility criteria. An additional 12 reports were excluded due to low methodological quality based on NOS and JBI assessments, while seven systematic reviews were excluded due to low AMSTAR 2 scores. Consequently, 22 studies identified through citation searching were included in the review. Together with the 45 studies identified through database searching, this resulted in a total of 67 studies included in the final review. The complete study selection process is presented in the PRISMA 2020 flow diagram in Figure 1.

### 3.3. Grey Literature Search Results

The grey literature search identified 181 potentially relevant records, of which 79 were removed as duplicates. Among the remaining 102 records, 62 could not be retrieved as full-text documents, leaving 40 records for eligibility assessment.

Following evaluation based on the predefined inclusion and exclusion criteria, 28 records were excluded, while 12 were retained as complementary evidence. These sources, consisting of doctoral theses, books, and other non-primary publications, were used exclusively for contextual interpretation and were not included among the 67 peer-reviewed studies forming the primary evidence synthesis.

### 3.4. Characteristics of Included Studies

The included studies presented heterogeneous designs, populations, diagnostic criteria, and imaging protocols. Most were observational, cross-sectional, or retrospective studies evaluating temporomandibular joint alterations in patients with TMD, TMJ osteoarthritis, internal derangement, or degenerative joint disease.

CBCT was the main imaging method used for assessing osseous changes, while MRI-based studies mainly focused on disc position, internal derangement, joint effusion, and soft-tissue pathology. Some studies combined CBCT with MRI, panoramic radiography, conventional CT, ultrasonography, Single-photon emission computed tomography/computed tomography (SPECT/CT), or arthroscopy.

The most frequently reported osseous findings were condylar flattening, erosion, sclerosis, osteophyte formation, cortical thinning or discontinuity, subchondral cystic changes, joint-space imbalance, condylar remodeling, and deformation of the glenoid fossa or articular eminence. Clinical correlations were variably reported and included pain, joint sounds, limited mouth opening, mandibular deviation, functional impairment, and occlusal instability.

Overall, the included studies provided a broad evidence base for identifying recurrent CBCT-detectable osseous patterns. However, most studies described these findings separately rather than integrating them into a reproducible structural severity scale, supporting the need for the proposed CBCT-based classification.

### 3.5. Methodological Quality and Risk of Bias Assessment

The Newcastle–Ottawa Scale was applied to observational studies, with the results presented in Table 3 [20]. The Joanna Briggs Institute critical appraisal tools were applied to cross-sectional, quasi-experimental, diagnostic accuracy, and randomized clinical studies, with the results presented in Table 4 [22]. AMSTAR 2 was applied to systematic reviews, with the results presented in Table 5 [21]. Risk-of-bias visualizations were generated using the robvis online visualization tool.

In the NOS assessment, most observational studies showed good methodological quality. Takaoka et al. [26], Bianchi et al. [27], Becenen Durmuş et al. [28], and the two studies by Emshoff et al. [29,30] obtained high scores because they used clearly defined study populations, standardized imaging assessment, appropriate statistical analysis, and, in several cases. Eisazadeh et al. [31], obtained a lower but still acceptable score because, although the study used standardized CBCT assessment and reported strong examiner reliability, it had limitations related to sample representativeness. Biplob and Prianka [32], was graded as 6 after score harmonization.

**Table 3:** Newcastle–Ottawa Scale assessment of observational studies.

Author, Year	C1	C2	C3	C4	C5	C6	C7	C8	TOTAL	Overall Assesment
Takaoka et al., 2022 [26]	1	1	1	2	1	1	1	1	9	Good
Bianchi et al., 2021 [27]	1	1	1	2	1	1	1	1	9	Good
Becenen Durmuş et al., 2025 [28]	1	1	1	2	1	1	1	1	9	Good
Emshoff et al., 2021 [29]	0	1	1	2	1	1	1	1	8	Good
Emshoff et al., 2021 (BMC) [30]	0	1	1	2	1	1	1	1	8	Good
Biplob & Prianka, 2025 [32]	1	1	1	2	0	0	0	1	6	Fair
Eisazadeh et al., 2021 [31]	0	1	0	2	1	1	1	1	7	Good

C1—Adequacy of case definition; C2—Representativeness of cases; C3—Selection of controls; C4—Definition of controls; C5—Comparability of cases and controls on the basis of design or analysis (maximum 2 stars); C6—Ascertainment of exposure; C7—Same method of ascertainment for cases and controls; C8—Non-response rate.

The JBI assessment showed that most clinical and imaging studies were suitable for inclusion, although many required cautious interpretation. Cross-sectional studies commonly scored 6/8. The strongest cross-sectional evidence was provided by studies such as Emshoff et al. [29], which obtained 8/8.

Among the randomized clinical studies assessed with the 13-item JBI checklist, Chen et al. [33], Rady et al. [34], and Ritto [35], obtained the highest scores, each reaching 11/13. These studies were graded favorably because they reported adequate randomization, comparable groups, reliable outcome assessment, and appropriate statistical analysis. Jia et al. [36] and Zheng et al. [37] also showed good quality, each scoring 10/13. Lower-scoring randomized studies, such as Sharma et al. [38] and Cömert Kılıç [39] were retained with caution because of unclear allocation procedures, incomplete blinding, or limited reporting of trial methodology. These limitations reduced confidence in treatment-related conclusions but did not eliminate their relevance for identifying clinical manifestations and therapeutic contexts associated with TMJ degeneration.

The quasi-experimental studies showed variable but generally acceptable methodological quality. Li et al. [40], Ren et al. [41], Musa et al. [42], and Tăut et al. [43], obtained high scores, mainly because they provided structured clinical or imaging follow-up and clear outcome assessment. Studies such as Sang et al. [44], Januzzi et al. [45], Ahmed et al. [46], and Hegab et al. [47], were interpreted more cautiously because of limitations related to non-randomized design, absence of a true control group, incomplete follow-up, or limited control of confounding factors.

Diagnostic accuracy studies were generally relevant for the imaging component of the review. Knezevic et al. [48], and Diniz de Lima et al. [49], obtained the strongest JBI diagnostic scores, each reaching 9/10. Gonzalez et al. [50], Mao et al. [51], and Dumanlı and Şeker [52], also showed acceptable quality, with scores of 8/10. Lower scores were mainly related to unclear patient selection, incomplete reporting of the reference standard, lack of independent interpretation between index test and reference test, or insufficient reporting of the time interval between diagnostic procedures.

**Table 4:** (**a**) Joanna Briggs Institute critical appraisal for cross-sectional studies. (**b**) Joanna Briggs Institute critical appraisal for Quasi-Experimental studies. (**c**) Joanna Briggs Institute critical appraisal for Diagnostic Test Accuracy Studies. (**d**) Joanna Briggs Institute critical appraisal for Randomized Controlled Trials.

(**a**)														
**Author, Year**	**Q1**	**Q2**	**Q3**	**Q4**	**Q5**	**Q6**	**Q7**	**Q8**						**Total Applicable Items**
Wu et al., 2025 [53]	Yes	Yes	Yes	Yes	No	No	Yes	Yes						6
Hoa et al., 2025 [54]	Yes	Yes	Yes	Yes	Unclear	No	Yes	Yes						6
Bertotti et al., 2025 [55]	Yes	Yes	Yes	Yes	Unclear	No	Yes	Yes						6
Topaloglu Yasan et al., 2023 [56]	Yes	Yes	Yes	Yes	Unclear	No	Yes	Yes						6
Mizuhashi et al., 2023 [57]	Unclear	Yes	Yes	Yes	Unclear	Yes	Yes	Yes						6
Fernández-Ferro et al., 2023 [58]	Yes	Yes	Yes	Yes	No	No	Yes	Yes						6
Yap et al., 2023 [59]	Yes	Yes	Yes	Yes	No	No	Yes	Yes						6
Collin et al., 2022 [60]	Yes	Yes	Yes	Yes	No	No	Yes	Yes						6
Tegnander et al., 2021 [61]	Yes	Yes	Yes	Yes	No	No	Yes	Yes						6
Schnabl et al., 2025 [62]	Yes	Yes	Yes	Yes	No	No	Yes	Yes						6
Koshal et al., 2021 [63]	Yes	Yes	Yes	Yes	No	No	Yes	Yes						6
Nina-Aguilar et al., 2025 [64]	Yes	Yes	Yes	Yes	No	No	Yes	Yes						6
Yulek et al., 2026 [65]	Yes	Yes	Yes	Yes	No	No	Yes	Yes						6
Paknahad et al., 2023 [66]	Yes	Yes	Yes	Yes	No	No	Yes	Yes						6
Chikkanna et al., 2026 [67]	Yes	Yes	Unclear	Yes	Yes	No	Yes	Yes						6
Jeon et al., 2021 [68]	Yes	Yes	Yes	Yes	No	No	Yes	Yes						6
Yildizer & Odabaşı, 2023 [69]	Yes	Yes	Yes	Yes	No	No	Yes	Yes						6
Görürgöz et al., 2023 [70]	Yes	Yes	Yes	Yes	Yes	Unclear	Yes	Yes						7
Jeon et al., 2023 [71]	Yes	Yes	Yes	Yes	No	No	Yes	Yes						6
Nayak et al., 2024 [72]	Yes	Yes	Yes	Yes	No	No	Yes	Yes						6
Ming et al., 2023 [73]	Yes	Yes	Yes	Yes	No	No	Yes	Yes						6
Hilgenberg-Sydney et al., 2024 [74]	Yes	Yes	Yes	Yes	Unclear	No	Yes	Yes						6
(**b**)														
**Author, Year**	**Q1**	**Q2**	**Q3**	**Q4**	**Q5**	**Q6**	**Q7**	**Q8**	**Q9**					**Total Applicable Items**
Li et al., 2024 [40]	Yes	Yes	Yes	Yes	No	Yes	Yes	Yes	Yes					8
Sang et al., 2024 [75]	Yes	Yes	Unclear	Yes	No	Unclear	Yes	Yes	Yes					6
Januzzi et al., 2025 [45]	Yes	Yes	Unclear	No	No	Yes	Yes	Yes	Yes					6
Ren et al., 2025 [41]	Yes	Yes	Yes	Yes	No	Yes	Yes	Yes	Yes					8
Musa et al., 2023 [42]	Yes	Yes	Yes	Yes	No	Yes	Yes	Yes	Yes					8
Ahmed et al., 2023 [46]	Yes	Yes	Yes	No	No	Yes	Yes	Yes	Yes					7
Hegab et al., 2021 [47]	Yes	Yes	Yes	No	No	Yes	Yes	Yes	Yes					7
Tăut et al., 2024 [43]	Yes	Yes	Yes	No	Yes	Yes	Yes	Yes	Yes					8
(**c**)														
**Author, Year**	**Q1**	**Q2**	**Q3**	**Q4**	**Q5**	**Q6**	**Q7**	**Q8**	**Q9**	**Q10**				**Total Applicable Items**
Ramesh et al., 2025 [76]	Unclear	Yes	Yes	No	No	Yes	Unclear	Yes	Yes	Yes				6
Millón Cruz et al., 2024 [77]	Yes	Yes	Yes	Yes	Unclear	Yes	Unclear	Unclear	Yes	Yes				7
Knezevic et al., 2023 [48]	Yes	Yes	Yes	Yes	Yes	Unclear	Yes	Yes	Yes	Yes				9
Diniz de Lima et al., 2022 [49]	Yes	Yes	Yes	Yes	Unclear	Yes	Yes	Yes	Yes	Yes				9
Gonzalez et al., 2021 [50]	Unclear	Yes	Yes	Yes	Unclear	Yes	Yes	Yes	Yes	Yes				8
Mao et al., 2025 [51]	Unclear	Unclear	Yes	Yes	Yes	Yes	Yes	Yes	Yes	Yes				8
Wojciechowska et al., 2024 [78]	Unclear	Yes	Yes	Unclear	Unclear	Yes	Yes	Yes	Yes	Yes				7
Dumanlı & Şeker, 2025 [52]	Unclear	Yes	Yes	Yes	Yes	Yes	Yes	Unclear	Yes	Yes				8
(**d**)														
**Author, Year**	**Q1**	**Q2**	**Q3**	**Q4**	**Q5**	**Q6**	**Q7**	**Q8**	**Q9**	**Q10**	**Q11**	**Q12**	**Q13**	**Total Applicable Items**
Chen et al., 2026 [33]	Yes	Yes	Yes	No	Yes	Yes	Yes	Yes	Unclear	Yes	Yes	Yes	Yes	11
Jia et al., 2024 [36]	Yes	Yes	Yes	No	No	Yes	Yes	Yes	Unclear	Yes	Yes	Yes	Yes	10
Zheng et al., 2026 [37]	Yes	Yes	Yes	Unclear	Unclear	Yes	Yes	Yes	Unclear	Yes	Yes	Yes	Yes	10
Farronato et al., 2025 [79]	Unclear	Unclear	Yes	No	No	Yes	Yes	Yes	Yes	Yes	Yes	Yes	Yes	9
Sharma et al., 2025 [38]	Yes	Unclear	Unclear	No	No	Unclear	Yes	Yes	Unclear	Yes	Yes	Unclear	Yes	6
Rady et al., 2022 [34]	Yes	Yes	Yes	No	No	Yes	Yes	Yes	Yes	Yes	Yes	Yes	Yes	11
Ritto et al., 2022 [35]	Yes	Yes	Yes	Yes	No	Yes	Yes	Yes	No	Yes	Yes	Yes	Yes	11
Yeladandi et al., 2024 [80]	Unclear	Unclear	Unclear	No	No	No	Yes	Yes	Yes	Yes	Yes	Yes	Yes	7
Comert Kılıç, 2021 [39]	Unclear	Unclear	Yes	No	No	Unclear	Yes	No	No	Yes	Yes	Yes	Yes	6

(**a**) Q1—Clarity of inclusion criteria; Q2—Detailed description of the study population and setting; Q3—Validity and reliability of exposure measurement; Q4—Objective and standardized measurement of the condition; Q5—Identification of confounding factors; Q6—Strategies for addressing confounding factors; Q7—Validity and reliability of outcome measurement; Q8—Appropriateness of statistical analysis. (**b**) Q1—Clarity of the cause-and-effect relationship; Q2—Similarity of participants between comparison groups; Q3—Similarity of care or treatment between groups apart from the intervention; Q4—Presence of a control group; Q5—Multiple measurements of the outcome before and after the intervention; Q6—Completeness of follow-up; Q7—Consistency of outcome measurement across groups; Q8—Reliability of outcome measurement; Q9—Appropriateness of statistical analysis. (**c**) Q1—Appropriateness of the patient sample; Q2—Consecutive or random recruitment of participants; Q3—Avoidance of inappropriate exclusions; Q4—Clear description of the index test; Q5—Validity of the reference standard; Q6—Independent interpretation of the index test and reference standard; Q7—Appropriate time interval between index test and reference standard; Q8—Use of the same reference standard for all participants; Q9—Inclusion of all participants in the analysis; Q10—Appropriateness of statistical analysis. (**d**) Q1—True randomization of participants; Q2—Concealment of allocation to treatment groups; Q3—Similarity of treatment groups at baseline; Q4—Blinding of participants; Q5—Blinding of treatment providers; Q6—Blinding of outcome assessors; Q7—Identical treatment of groups apart from the intervention of interest; Q8—Completeness of follow-up; Q9—Analysis according to randomized groups/intention-to-treat principle; Q10—Consistency of outcome measurement across groups; Q11—Reliability of outcome measurement; Q12—Appropriateness of statistical analysis; Q13—Appropriateness of the trial design and consideration of deviations from a standard randomized controlled trial design.

The AMSTAR 2 assessment showed that the included systematic reviews had low or critically low overall confidence. Mehta et al. [81], Ronsivalle et al. [82], Wu et al. [83], Sang et al. [44], and Farook and Dudley [84] were rated as low confidence. These reviews generally had clear research questions, structured search strategies, and risk-of-bias assessment, but were downgraded because of incomplete reporting of funding sources, limited duplicate extraction, incomplete publication-bias assessment, or limited integration of risk of bias into the interpretation of results. Ehrmann et al. [85], Alvear Miquilena et al. [86], Minervini et al. [87], and Dygas et al. [88], were rated as critically low confidence, mainly because of more important limitations in search strategy, reporting of excluded studies, risk-of-bias assessment, or methodological transparency. Therefore, systematic reviews were used mainly to contextualize the field and identify gaps, rather than as the strongest source of evidence for developing the proposed CBCT-based scale.

**Table 5:** AMSTAR 2 assessment of systematic reviews and evidence syntheses.

Author, Year	Q2:	Q4:	Q7:	Q9:	Q11:	Q13:	Q15:	Overall Rating
Ehrmann et al., 2024 [85]	Yes	No	No	No	No Meta-analysis/NA	No	No Meta-analysis/NA	Critically Low
Mehta et al., 2026 [81]	Yes	Yes	Yes	Yes	Yes	Yes	Yes	Low
Alvear Miquilena et al., 2024 [86]	Yes	Yes	Partial Yes	Partial Yes	Partial Yes	Partial Yes	No	Critically Low
Ronsivalle et al., 2024 [82]	Yes	Partial Yes	Yes	Yes	Yes	Partial Yes	No/insufficient studies	Low
Minervini et al., 2023 [87]	Yes	Partial Yes	No	Yes	Yes	Partial Yes	No/insufficient studies	Critically Low
Wu et al., 2021 [83]	Yes	Yes	Yes	Yes	No Meta-analysis/NA	Yes	No Meta-analysis/NA	Low
Sang et al., 2024 (MRI SR) [44]	Yes	Yes	Yes	Yes	No Meta-analysis/NA	Yes	No Meta-analysis/NA	Low
Dygas et al., 2022 [88]	No	Yes	No	Yes	No Meta-analysis/NA	Partial Yes	No Meta-analysis/NA	Critically Low
Farook & Dudley, 2023 [84]	Yes	Yes	Partial Yes	Yes	No Meta-analysis/NA	Yes	No Meta-analysis/NA	Low

Q2—Protocol established prior to conducting the review; Q4—Comprehensive literature search strategy; Q7—Provision of a list of excluded studies with justification; Q9—Satisfactory risk-of-bias assessment technique; Q11—Appropriateness of meta-analytical methods, when applicable; Q13—Consideration of risk of bias when interpreting the results; Q15—Investigation of publication bias, when applicable.

**Figure 2:**
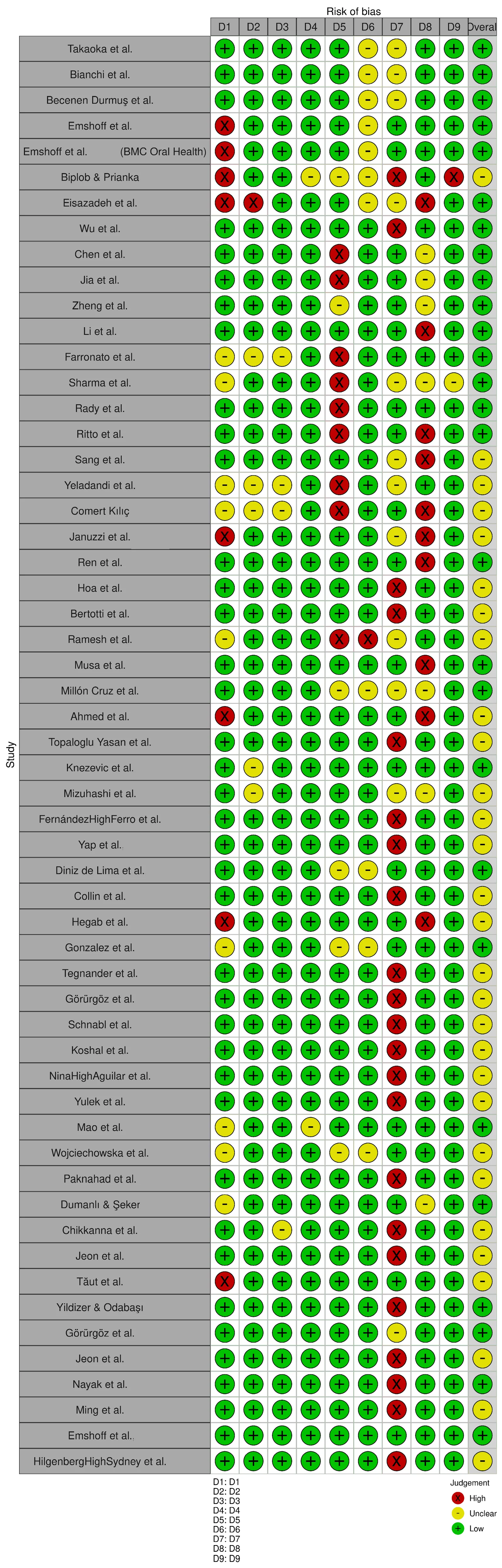
Traffic-light risk-of-bias plot of the included studies [26,27,28,29,30,31,32,33,34,35,36,37,38,39,40,41,42,43,44,45,46,47,48,49,50,51,52,54,55,56,57,58,59,60,61,62,63,64,65,66,67,68,69,70,71,72,73,74,76,77,78,79,80,83,89].

**Figure 3:**
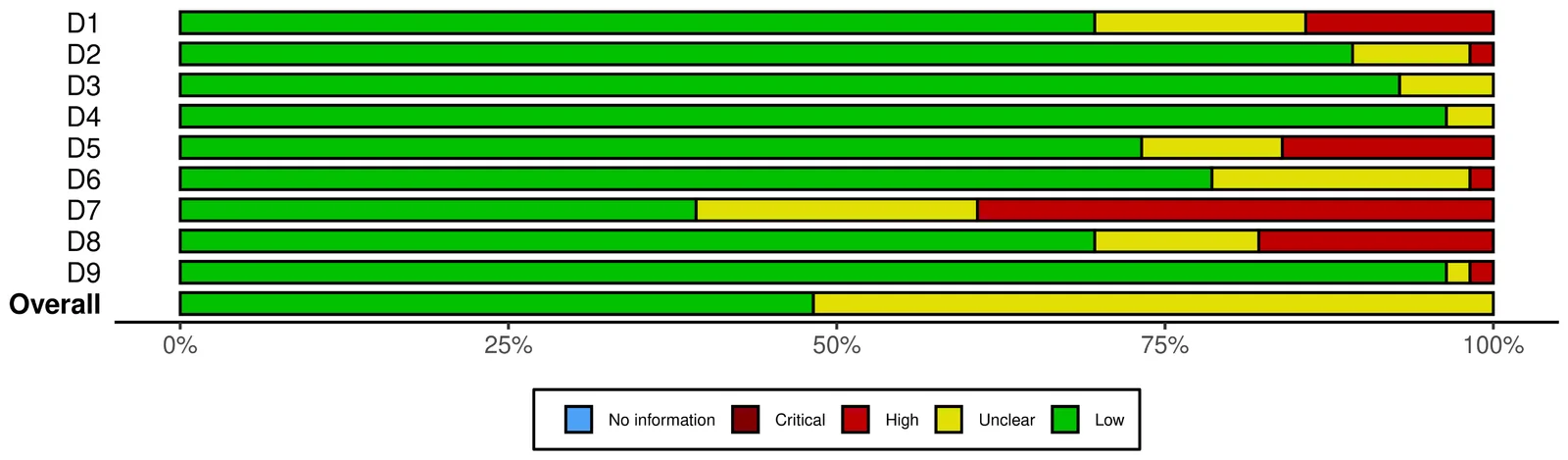
Weighted summary risk-of-bias plot of the included studies.

Risk-of-bias visualizations were generated using the robvis online visualization tool. The traffic-light plot is presented in Figure 2, while the weighted summary plot is presented in Figure 3. Because the included studies had different designs and were assessed with different tools, the visual synthesis was based on common methodological domains, including patient selection, definition of eligibility criteria, quality of the imaging protocol, objectivity of outcome assessment, examiner blinding or calibration, reporting of inter- or intra-observer reliability, control of confounding factors, completeness of data, and appropriateness of statistical analysis.

Overall, the risk-of-bias assessment showed that the included evidence was usable, but heterogeneous. The strongest studies were those with clearly defined TMD or TMJ osteoarthritis populations, standardized CBCT or MRI protocols, objective assessment of osseous changes, examiner blinding, reliability testing, and appropriate statistical analysis. The main sources of bias across the included literature were single-center recruitment, retrospective design, limited control of confounders, incomplete reporting of imaging protocols, and absence of inter- or intra-observer reliability. In this context, lower risk of bias referred mainly to better transparency in patient selection, imaging assessment, reproducibility, and statistical handling, rather than to the complete absence of bias.

### 3.6. Literature Search Findings

#### 3.6.1. Main TMJ Bone Changes Found in the Studies

Across the included studies, temporomandibular joint degeneration was most consistently described through recurrent osseous changes involving the mandibular condyle, cortical bone, subchondral bone, glenoid fossa, articular eminence, and joint-space relationships. CBCT-based studies. The main osseous findings extracted from each study are summarized in Table 6.

#### 3.6.2. Evolution of TMJ Bone Changes

Based on the recurrent findings extracted from the included studies, TMJ osseous alterations could be organized into an inferred radiologic severity sequence. Early adaptive changes were mainly represented by mild condylar asymmetry, altered condylar position, joint-space imbalance, and discrete condylar flattening with preserved cortical continuity, as reported in studies such as Paknahad et al. [66], Bianchi et al. [27], Görürgöz et al. [70], and Schnabl et al. [62]. More established structural remodelling was characterized by evident flattening, cortical thinning, subchondral sclerosis, osteophyte formation, and dimensional changes of the condylar head. Advanced structural degeneration was represented by marked condylar deformation, crateriform defects, severe joint-space reduction, remodelling of the glenoid fossa or articular eminence, and bone-to-bone contact.

The radiological findings and their structural severity associations, as derived from the included literature, are summarized in Table 7. These associations should not be interpreted as a universally applicable chronological sequence, since individual alterations may coexist or occur independently. The study-level evidence supporting this sequence is summarized in Table 7.

#### 3.6.3. Link Between TMJ Bone Changes and Clinical Symptoms

The association between osseous TMJ alterations and clinical manifestations was reported inconsistently across the included studies. Most of the 67 studies reported osseous changes and clinical symptoms in parallel, but only a limited number formally tested or reported a direct statistical correlation between a specific bone alteration and a specific clinical sign. The study-level associations between individual osseous changes and clinical manifestations are summarized in Table 8.

Table 8 summarizes reported associations between radiological findings and clinical manifestations. Findings supported by direct statistical comparisons are distinguished from associations described narratively or summarized in systematic reviews. Given the heterogeneity of study populations, diagnostic criteria, imaging modalities, and statistical methods, these findings should not be interpreted as evidence of a consistent or causal relationship between individual osseous alterations and clinical symptoms.

#### 3.6.4. Existing TMJ Classification Systems

Several classification systems were identified across the included studies, but most were designed for clinical diagnosis or treatment stratification rather than for CBCT-based staging of osseous degeneration. Such clinical diagnostic tools are the DC/TMD and RDC/TMD.

MRI-based classifications were also frequently represented. Wilkes staging was used in studies such as Ritto et al. [35], Topaloglu Yasan et al. [56], Fernández-Ferro et al. [58], and Cömert Kılıç [39], while Piper staging was reported in Tegnander et al. [61]. The Hegab MRI-based classification, described by Hegab et al. [47] also proposed stages of internal derangement and linked advanced stages with more complex therapeutic approaches.

A smaller number of studies used CBCT-oriented or osseous-focused classifications. Emshoff et al. [29] proposed a composite CBCT-based subchondral bone alteration score, while Becenen Durmuş et al. [28] classified joints into no, early, and late degenerative joint disease groups. Other CBCT studies generally described these findings individually rather than integrating them into a reproducible CBCT staging system. Overall, the classification systems identified in the review supported the clinical relevance of imaging-based TMJ assessment, but also confirmed the absence of a simple CBCT-based scale dedicated specifically to staging osseous structural severity. The main classification systems, imaging methods, stages, osseous findings, clinical correlations, and treatment implications are summarized in Table 9.

### 3.7. Certainty of Evidence for the Main Review Findings

A GRADE-informed assessment was used to evaluate the certainty of evidence for the main review findings, rather than for each study individually. The complete assessment is presented in Table 10.

The evidence was considered sufficient to support the development of a CBCT-based staging system, but not strong enough to define strict prognostic or therapeutic rules based on individual osseous findings.

### 3.8. Proposal of the New CBCT-Based TMJ Scale

Based on the findings of the literature review, a new four-stage CBCT-based scale was proposed for grading osseous TMJ alterations in patients with TMDs. Unlike MRI-based classifications, which mainly focus on disc position and internal derangement, the proposed scale focuses specifically on, mostly, osseous parameters visible on CBCT.

The complete structure of the proposed CBCT-based scale is presented in Table 11.

Joint-space narrowing should be interpreted alongside osseous morphological alterations and should not independently determine progression to a higher stage.

The proposed stages are primarily based on the extent of osseous morphological alterations, while condylar position and joint-space relationships are considered supportive findings. Individual radiological signs may coexist across the proposed stages, and their presence alone does not necessarily justify assignment to a more advanced category. In cases presenting overlapping characteristics, interpretation should consider the overall pattern and extent of structural involvement. Definitive rules for stage assignment, including mandatory criteria, quantitative thresholds, and the handling of discordant findings, remain to be established through dedicated validation studies.

To further clarify the specific contribution of the proposed classification, Table 12 compares existing CBCT-based scoring and classification systems regarding their anatomical criteria, grading principles, validation status, and reliability. This comparison highlights the broader anatomical scope of the proposed framework while acknowledging that its operational thresholds and reproducibility remain to be established.

## 4. Discussion

The present study identified recurrent CBCT-detectable bone changes of the temporomandibular joint and organized them into a preliminary four-stage severity framework.

This classification should not be interpreted as another diagnostic system for temporomandibular disorders. Instead, it was designed to stage the osseous component of TMJ involvement by assessing the changes that appear at the TMJ level. Therefore, the proposed scale complements existing diagnostic frameworks rather than replacing them. Clinical diagnostic systems remain essential for identifying TMD subtypes, while MRI remains necessary for evaluating soft-tissue involvement [47,55]. The contribution of the present scale is that it provides a structured and reproducible method for grading CBCT-visible osseous severity, which may be useful in dental, orthodontic, prosthetic and maxillofacial settings, where CBCT is frequently available.

The proposed CBCT-based scale differs from existing TMJ classification systems because it focuses specifically on osseous structural severity. DC/TMD remains essential for clinical diagnosis and for identifying TMD subgroups, but it is not designed as an imaging-based scale [5]. Wilkes and Piper classifications [61] are important for staging internal derangement and disc-condyle relationships. However, they rely mainly on MRI-based evaluation and emphasize disc position, disc deformation, adhesions, perforation, inflammatory soft-tissue changes, and late joint degeneration [12].

Furthermore, the proposed classification should not be interpreted as an independent determinant of treatment selection, as therapeutic decisions require comprehensive clinical evaluation, including symptoms, functional impairment, and patient-specific factors. The relationship between the proposed structural stages and treatment outcomes remains to be established through prospective clinical studies.

Therefore, the proposed scale should not be interpreted as superior to MRI-based systems for disc-related pathology. Its advantage is different: it provides a practical and reproducible method for staging CBCT-visible bone changes in settings where CBCT is frequently available, such as dental, orthodontic, prosthetic, oral surgery, and maxillofacial practice. In this sense, the scale complements other classifications by adding a structured osseous severity component [14,27].

Unlike previous CBCT-based classifications, such as the subchondral bone alteration grading system proposed by Emshoff et al. [29], which primarily evaluates individual degenerative findings and their combinations, our classification integrates additional anatomical parameters, including condylar dimensions, spatial relationships within the joint, cortical integrity, and morphological changes of the glenoid fossa and articular eminence. These parameters are organized into four stages reflecting increasing structural severity rather than simply the presence or combination of individual findings. This approach may provide a more comprehensive assessment of osseous TMJ involvement and facilitate standardized radiological reporting and clinical monitoring, although its clinical utility and reproducibility remain to be validated.

The proposed classification is intended exclusively to describe the severity of CBCT-detectable osseous alterations and does not provide treatment recommendations. Therapeutic decisions should remain based on clinical diagnosis, symptoms, functional impairment, and individual patient factors. The potential clinical applicability of this classification, including its role in treatment planning, will be investigated in future validation studies. Stage I represents normal or near-normal osseous morphology. In this stage, when symptoms are absent or mild, clinical monitoring, patient education, conservative care, and periodic reassessment may be sufficient. Stage II suggests early positional or adaptive changes. These findings may justify functional assessment, monitoring, physiotherapy, behavioral management, and occlusal stabilization when clinically indicated.

Stage III reflects established osseous remodeling. In symptomatic patients, this stage may require a more active conservative or minimally invasive approach, including pain control, anti-inflammatory strategies, splint therapy, physiotherapy, functional rehabilitation, and, when appropriate, intra-articular or arthrocentesis-based interventions. Stage IV represents advanced structural degeneration. These cases may require multidisciplinary assessment, MRI evaluation when disc or soft-tissue pathology is suspected, advanced pain and functional rehabilitation, and possible oral and maxillofacial surgical consultation.

A 2021 systematic review by Wu et al. [83] showed that the association between CBCT-detected osseous changes and clinical symptoms in TMJ degenerative disease is heterogeneous, while a 2025 study [53] found a significant association mainly between clicking and osteophytes, but not between CBCT findings and muscle or joint pain.

The main strength of this study is the practical design of the classification. The resulting system is simple, stage-based, and clinically understandable, which may facilitate its use in dental, orthodontic, prosthetic, radiologic, and maxillofacial settings. Previous CBCT-based classifications, including those proposed by Emshoff, Bianchi, Yap, Nayak, and Koyama, have already introduced structured approaches to grading or categorizing osseous TMJ alterations. The present framework differs primarily by integrating condylar dimensions, joint-space relationships, cortical integrity, and glenoid fossa/articular eminence morphology into four proposed structural severity stages. However, unlike established scoring approaches, the proposed system has not yet undergone threshold definition, reliability testing, or external validation. However, most of these approaches focus on identifying only individual osseous findings or distinguishing osteoarthritic from non-osteoarthritic changes.

The proposed classification has several limitations. First, CBCT assesses only the osseous components of the TMJ and cannot directly evaluate the articular disc, joint effusion, retrodiscal tissues, or inflammatory soft-tissue changes. Furthermore, joint-space relationships may be influenced by disc position and other factors, and should therefore not be interpreted as independent indicators of osseous degeneration. Clinical symptoms may also correlate poorly with structural alterations, given the multifactorial nature of TMD-related pain and dysfunction.

Regarding the review methodology, the inability to retrieve 45 full-text reports identified through database searching (46.4%) and 117 through supplementary citation searching (60.0%) represents a potential source of selection bias, as relevant evidence may have been overlooked. This limitation may have affected the comprehensiveness of the literature synthesis underlying the proposed classification.

Additionally, the proposed four-stage framework remains conceptual, as standardized operational criteria, quantitative thresholds, and inter- and intra-examiner reliability have not yet been established. The stages reflect hypothesized differences in structural severity rather than a validated chronological sequence of disease progression. Consequently, the classification should not independently guide clinical decisions or be considered suitable for routine implementation without further validation.

Future studies should establish standardized staging criteria, address physiological and age-related variations, evaluate discordant or bilateral findings, and assess reproducibility in representative patient populations. Larger multicenter and longitudinal investigations, including comparisons with MRI findings, are necessary to establish external validity, clinical utility, and potential associations with disease progression or treatment outcomes. AI-assisted CBCT analysis may also support future improvements in measurement standardization and reproducibility. Importantly, because CBCT involves ionizing radiation, its use should remain restricted to clinically justified examinations rather than routine screening of all patients with TMD.

In addition, clinical symptoms may not always correlate directly with osseous changes, because pain and dysfunction in TMD are multifactorial. Finally, because CBCT involves ionizing radiation, the proposed scale should be applied only when CBCT examination is clinically justified and not as a screening tool for all patients with TMD.

Future studies should validate the proposed scale clinically, followed by validation in larger, multicenter cohorts. It would also be valuable to compare the changes seen on CBCT with MRI findings. Longitudinal studies are also needed to determine whether the proposed stages truly reflect disease progression over time and whether specific stages can predict response to conservative, minimally invasive, or surgical treatment.

Another important direction is AI-assisted CBCT analysis. Automated assessment of joint spaces, condylar dimensions, cortical defects, and bone-to-bone contact may improve reproducibility and support integration of the scale into clinical imaging software.

## 5. Conclusions

The main finding of this study is that CBCT-detectable osseous alterations of the temporomandibular joint can be organized into a preliminary four-stage structural severity framework. The proposed classification integrates individual bone changes into a structured system intended to facilitate the description and assessment of TMJ osseous involvement.

The scale may provide a practical basis for estimating structural severity using an imaging technique that is widely accessible in dental radiology settings. However, the proposed stages reflect hypothesized differences in morphological severity rather than a validated chronological sequence of disease progression and should not independently determine treatment selection.

Although the classification integrates multiple anatomical parameters, it remains conceptual, as quantitative thresholds, reproducibility, and clinical utility have not yet been established. Joint-space and positional alterations should be interpreted as supportive findings rather than independent indicators of osseous degeneration. Further studies assessing inter- and intra-examiner reliability, external validity, longitudinal progression, and clinical applicability are necessary before routine clinical implementation.

## Figures and Tables

**Table 1:** Database-specific search strategy used to identify studies on CBCT-detectable osseous changes of the temporomandibular joint in TMD.

PubMed
((“Temporomandibular Joint Disorders”[MeSH] OR “Temporomandibular Joint”[MeSH] OR “temporomandibular disorders”[Title/Abstract] OR “temporomandibular joint disorders”[Title/Abstract] OR TMD[Title/Abstract] OR TMJ[Title/Abstract]) AND (“Cone-Beam Computed Tomography”[MeSH] OR CBCT[Title/Abstract] OR “cone beam computed tomography”[Title/Abstract]) AND (“Osteoarthritis”[MeSH] OR “degenerative joint disease”[Title/Abstract] OR “osseous changes”[Title/Abstract] OR “bony changes”[Title/Abstract] OR “condylar remodeling”[Title/Abstract] OR flattening[Title/Abstract] OR erosion[Title/Abstract] OR sclerosis[Title/Abstract] OR osteophyte[Title/Abstract] OR “cortical thinning”[Title/Abstract] OR “joint space”[Title/Abstract]) AND (“clinical correlation”[Title/Abstract] OR “clinical manifestations”[Title/Abstract] OR symptoms[Title/Abstract] OR pain[Title/Abstract] OR “limited mouth opening”[Title/Abstract] OR “mandibular deviation”[Title/Abstract])) AND english[Language] AND (“1 January 2021”[Date—Publication]: “31 March 2026”[Date—Publication])
**Embase**
(‘temporomandibular joint disorder’/exp OR ‘temporomandibular joint’/exp OR ‘temporomandibular disorder*’:ti,ab OR ‘temporomandibular joint disorder*’:ti,ab OR tmd:ti,ab OR tmj:ti,ab) AND (‘cone beam computed tomography’/exp OR cbct:ti,ab OR ‘cone beam computed tomography’:ti,ab) AND (‘osteoarthritis’/exp OR ‘degenerative joint disease’:ti,ab OR ‘osseous change*’:ti,ab OR ‘bony change*’:ti,ab OR ‘condylar remodeling’:ti,ab OR flattening:ti,ab OR erosion:ti,ab OR sclerosis:ti,ab OR osteophyte*:ti,ab OR ‘cortical thinning’:ti,ab OR ‘joint space’:ti,ab) AND (‘clinical correlation’:ti,ab OR ‘clinical manifestation*’:ti,ab OR symptom*:ti,ab OR pain:ti,ab OR ‘limited mouth opening’:ti,ab OR ‘mandibular deviation’:ti,ab) AND [english]/lim AND [2021–2026]/py
**Cochrane Library**
(“temporomandibular disorder” OR “temporomandibular joint disorder” OR TMD OR TMJ) AND (CBCT OR “cone beam computed tomography”) AND (“osseous changes” OR “bony changes” OR “degenerative joint disease” OR osteoarthritis OR “condylar remodeling” OR flattening OR erosion OR sclerosis OR osteophyte OR “cortical thinning” OR “joint space”) AND (“clinical correlation” OR “clinical manifestations” OR symptoms OR pain OR “limited mouth opening” OR “mandibular deviation”)

**Table 2:** Database-specific search strategy used to identify existing classification systems for temporomandibular disorders and temporomandibular joint internal derangement.

PubMed
((“Temporomandibular Joint Disorders”[MeSH] OR “Temporomandibular Joint”[MeSH] OR “temporomandibular disorders”[Title/Abstract] OR “temporomandibular joint disorders”[Title/Abstract] OR TMD[Title/Abstract] OR TMJ[Title/Abstract]) AND (classification[Title/Abstract] OR staging[Title/Abstract] OR grading[Title/Abstract] OR scale[Title/Abstract] OR “diagnostic criteria”[Title/Abstract]) AND (“RDC/TMD”[Title/Abstract] OR “Research Diagnostic Criteria for Temporomandibular Disorders”[Title/Abstract] OR “DC/TMD”[Title/Abstract] OR “Diagnostic Criteria for Temporomandibular Disorders”[Title/Abstract] OR Wilkes[Title/Abstract] OR Piper[Title/Abstract] OR “internal derangement”[Title/Abstract] OR “disc displacement”[Title/Abstract]) AND (“magnetic resonance imaging”[MeSH] OR MRI[Title/Abstract] OR “magnetic resonance imaging”[Title/Abstract] OR imaging[Title/Abstract])) AND english[Language] AND (“1 January 2021”[Date—Publication]: “31 March 2026”[Date—Publication])
**Embase**
(‘temporomandibular joint disorder’/exp OR ‘temporomandibular joint’/exp OR ‘temporomandibular disorder*’:ti,ab OR ‘temporomandibular joint disorder*’:ti,ab OR tmd:ti,ab OR tmj:ti,ab) AND (classification:ti,ab OR staging:ti,ab OR grading:ti,ab OR scale:ti,ab OR ‘diagnostic criteria’:ti,ab) AND (‘RDC/TMD’:ti,ab OR ‘Research Diagnostic Criteria for Temporomandibular Disorders’:ti,ab OR ‘DC/TMD’:ti,ab OR ‘Diagnostic Criteria for Temporomandibular Disorders’:ti,ab OR Wilkes:ti,ab OR Piper:ti,ab OR ‘internal derangement’:ti,ab OR ‘disc displacement’:ti,ab) AND (‘magnetic resonance imaging’/exp OR mri:ti,ab OR ‘magnetic resonance imaging’:ti,ab OR imaging:ti,ab) AND [english]/lim AND [2021–2026]/py
**Cochrane Library**
(“temporomandibular disorder” OR “temporomandibular joint disorder” OR TMD OR TMJ) AND (classification OR staging OR grading OR scale OR “diagnostic criteria”) AND (“RDC/TMD” OR “Research Diagnostic Criteria for Temporomandibular Disorders” OR “DC/TMD” OR “Diagnostic Criteria for Temporomandibular Disorders” OR Wilkes OR Piper OR “internal derangement” OR “disc displacement”) AND (MRI OR “magnetic resonance imaging” OR imaging)

**Table 6:** Main osseous temporomandibular joint alterations identified in the included imaging studies.

Author, Year	Imaging Method	Osseous Alterations
Wu et al., 2025 [53]	CBCT	Condylar flattening Erosion Subchondral sclerosis Osteophyte formation Subchondral cysts/pseudocysts Joint-space narrowing
Chen et al., 2026 [33]	CBCT	Condylar remodeling
Jia et al., 2024 [36]	CBCT	Condylar flattening Erosion Subchondral sclerosis Osteophyte formation
Zheng et al., 2026 [37]	CBCT	Condylar hypoplasia or resorption
Li et al., 2024 [40]	CBCT	Condylar remodeling Condylar hypoplasia or resorption
Farronato et al., 2025 [79]	CBCT	Condylar remodeling
Sharma et al., 2025 [38]	JVA only; no CBCT, MRI, or panoramic imaging used for primary assessment.	Condylar remodeling
Takaoka et al., 2022 [26]	MRI	Articular eminence remodeling
Bianchi et al., 2021 [27]	CBCT	Condylar flattening Erosion Subchondral sclerosis Osteophyte formation Subchondral cysts/pseudocysts
Comert Kılıç, 2021 [39]	CBCT	Condylar remodeling
Januzzi et al., 2025 [45]	CBCT + MRI preoperatively; CBCT/tomographic follow-up; ultrasonography for procedural guidance	Condylar remodeling
Emshoff et al., 2021 [29]	CBCT	Condylar flattening Erosion Subchondral sclerosis Osteophyte formation Subchondral cysts/pseudocysts Condylar hypoplasia or resorption
Ren et al., 2025 [41]	CBCT	Condylar asymmetry
Becenen Durmuş et al., 2025 [28]	Ultrasonography (USG); no CBCT/MRI primary imaging	Osteophyte formation
Musa et al., 2023 [42]	CBCT	Condylar dimensional reduction
Hilgenberg-Sydney et al., 2024 [74]	CBCT	Erosion Osteophyte formation Joint-space narrowing
Millón Cruz et al., 2024 [77]	MRI with arthroscopy as reference standard	Osteophyte formation
Ronsivalle et al., 2024 [82]	Mixed, depending on included studies: DC/TMD clinical assessment, MRI, CBCT, and radiographic assessment; no primary CBCT acquisition performed by the review itself.	Condylar flattening Erosion
Topaloglu Yasan et al., 2023 [56]	MRI	Condylar flattening Subchondral sclerosis Osteophyte formation
Mizuhashi et al., 2023 [57]	MRI	Condylar deformation
Yap et al., 2023 [59]	CBCT	Erosion Subchondral sclerosis Osteophyte formation Subchondral cysts/pseudocysts
Diniz de Lima et al., 2022 [49]	Infrared thermography (IT) with artificial intelligence/machine learning; not CBCT, MRI, or panoramic radiography.	Condylar flattening Erosion Subchondral sclerosis Osteophyte formation
Collin et al., 2022 [60]	CBCT	Condylar flattening Erosion Glenoid fossa remodeling
Emshoff et al., 2021 [29]	MRI	Erosion
Emshoff et al., 2021 (BMC Oral Health) [30]	MRI	Subchondral sclerosis
Wu et al., 2021 [83]	CBCT	Erosion
Sang et al., 2024 (MRI SR) [44]	MRI	Erosion
Görürgöz et al., 2023 [70]	CBCT	Condylar flattening Erosion Subchondral sclerosis Osteophyte formation Subchondral cysts/pseudocysts
Schnabl et al., 2025 [62]	CBCT	Condylar flattening Erosion Subchondral sclerosis Osteophyte formation Subchondral cysts/pseudocysts Glenoid fossa remodeling Condylar hypoplasia or resorption
Semerci & Günen Yılmaz, 2024 [90]	CBCT	Condylar flattening Erosion Subchondral sclerosis Osteophyte formation Subchondral cysts/pseudocysts Condylar hypoplasia or resorption Loose bodies/calcified intra-articular bodies
Biplob & Prianka, 2025 [32]	CBCT; digital OPG also used.	Condylar flattening Erosion Subchondral sclerosis Osteophyte formation Subchondral cysts/pseudocysts
Koshal et al., 2021 [63]	CT	Condylar flattening Erosion Subchondral sclerosis Osteophyte formation Subchondral cysts/pseudocysts Articular eminence remodeling
Nina-Aguilar et al., 2025 [64]	CBCT	Condylar flattening Erosion Subchondral sclerosis Osteophyte formation Subchondral cysts/pseudocysts
Yulek et al., 2026 [65]	CBCT	Condylar flattening Erosion Subchondral sclerosis Osteophyte formation
Mao et al., 2025 [51]	CBCT	Erosion Subchondral sclerosis Osteophyte formation Subchondral cysts/pseudocysts
Wojciechowska et al., 2024 [78]	CBCT and ultrasonography	Erosion Osteophyte formation
Paknahad et al., 2023 [66]	CBCT	Condylar flattening Erosion Subchondral sclerosis Osteophyte formation Subchondral cysts/pseudocysts
Dumanlı & Şeker, 2025 [52]	CBCT + panoramic radiography + ultrasonography (USG)	Condylar flattening Erosion Subchondral sclerosis Osteophyte formation Subchondral cysts/pseudocysts Loose bodies/calcified intra-articular bodies
Chikkanna et al., 2026 [67]	Conventional CT + orthopantomography screening	Condylar flattening Subchondral sclerosis Osteophyte formation
Jeon et al., 2021 [71]	CBCT and MRI	Condylar flattening Erosion Subchondral sclerosis Osteophyte formation
Tăut et al., 2024 [43]	CBCT	Condylar flattening Erosion Subchondral cysts/pseudocysts Condylar remodeling
Yildizer & Odabaşı, 2023 [69]	CBCT	Erosion Subchondral sclerosis Osteophyte formation Glenoid fossa remodeling
Ming et al., 2023 [73]	MRI (3.0T PDWI; HNCC vs. wireless detector-HNCC)	Erosion Subchondral sclerosis Osteophyte formation Subchondral cysts/pseudocysts

**Table 7:** Radiologic descriptors of TMJ osseous alterations and their proposed structural severity associations.

Stage	Osseous Alteration	Description/Mechanism	Typical Imaging Correlate
Early	Subchondral sclerosis [62,66,71,83]	Increased bone density beneath the articular surface as an early adaptive/reactive response to altered or excessive loading, before structural breakdown occurs [27].	Increased radiodensity on CBCT/CT beneath the condylar cortex
Early	Condylar flattening [29,62,70,83]	Loss of the normal convex contour of the condyle; an early morphologic adaptation to chronic mechanical loading, often preceding overt cortical breakdown [62,63].	Flat or slightly concave condylar contour on CBCT/MRI
Early	Surface irregularity [47]	Irregular or uneven cortical outline appearing as the smooth condylar surface begins to lose its regular architecture [47,71,73]	Roughened/irregular cortical outline
Early-Intermediate	Cortical thinning [72]	Reduction in cortical bone thickness, suggesting early structural weakening as remodeling shifts from adaptive to degenerative [71,72].	Thin, attenuated cortical line on CBCT/CT
Intermediate	Osteophyte formation [27,29,36,65,67,69]	Marginal bony outgrowths that develop as the joint attempts further adaptive remodeling in response to persistent abnormal loading [26,32,62].	Beak-like or rounded bony marginal projections
Intermediate	Cortical discontinuity/cortical defect [40,45]	Interruption of the cortical outline, marking the transition from adaptive remodeling to active degenerative damage [45].	Break/gap in the cortical line
Intermediate	Erosion [27,29,36,59,60,74,75,83]	Localized loss of cortical/subcortical bone; an active degenerative change once cortical integrity has been breached [27,59].	Focal radiolucent surface defect
Intermediate	Subchondral cysts/pseudocysts [27,29,43,66,70,72,73]	Radiolucent defects beneath the cortical surface associated with established degenerative joint disease [62,70].	Well- or ill-defined radiolucency beneath the cortex
Advanced	Crateriform defects [5,45]	Deeper, concave surface defects reflecting more advanced structural destruction of the condylar head [45].	Bowl-shaped erosive defect on CBCT/CT/MRI
Advanced	Condylar remodelling	Broader change in condylar shape/architecture from the combined effect of adaptive and degenerative processes accumulating over time [37,40].	Altered overall condylar morphology vs. normal anatomy
Advanced	Condylar dimensional reduction [42]	Reduced condylar height, width, or volume as bone loss outweighs any compensatory remodeling [37,42].	Smaller condylar dimensions on serial/comparative imaging
Advanced	Condylar asymmetry [42]	Differences in shape, size, height, or position between right and left condyles, reflecting unilateral or asymmetric disease progression [42].	Side-to-side morphometric differences
Advanced	Condylar hypoplasia or resorption [29,32,37,45,62]	Reduced condylar development or progressive loss of condylar bone volume, often a late consequence of long-standing degeneration [29,37,45].	Markedly diminished condylar volume/height
Advanced	Condylar deformation [60,69]	Marked, often global alteration of normal condylar morphology, typically representing an advanced stage of joint breakdown [60,69].	Grossly distorted condylar shape
Advanced	Joint-space narrowing [67,71,72,83,90]	Reduction of the superior, anterior, or posterior joint space as articular bone and disc tissue are progressively lost [72,83].	Decreased joint space width on CBCT/MRI
Advanced	Joint-space widening or imbalance [42]	Abnormal condylar position within the fossa producing asymmetric anterior/posterior/superior spaces, reflecting positional/structural instability [42,46].	Uneven anterior vs. posterior/superior joint space
End-stage	Bone-to-bone contact [32]	Severe reduction or complete loss of joint space indicating advanced/end-stage degeneration [32].	Direct apposition of condyle and fossa with no visible joint space
End-stage/Secondary	Glenoid fossa remodeling [43]	Flattening, sclerosis, or deformation of the mandibular fossa as the degenerative process extends to the opposing joint surface [43].	Altered fossa contour/density on CBCT/CT/MRI
End-stage/Secondary	Articular eminence remodeling [70]	Change in the contour, inclination, or cortical structure of the articular eminence, usually a late, secondary adaptation [70].	Altered eminence inclination/cortical outline
End-stage	Loose bodies/calcified intra-articular bodies [52,90]	Detached calcified fragments within the joint space; an uncommon but recognized finding in advanced degenerative disease.	Discrete calcified densities within the joint space [52,90]

**Table 8:** Relationship between imaging-detected TMJ osseous changes and clinical manifestations.

Author, Year	Osseous Alteration	Corresponding Clinical Manifestation
Wu et al., 2025 [53]	Osteophyte formation	TMJ clicking was significantly associated with bilateral osteophyte formation; no significant association was found between joint/muscle pain or crepitus and osteophytes.
Emshoff et al., 2021 [29]	Erosion	Painful closed lock/arthralgia with disc displacement without reduction was most clearly associated with condylar erosion; TMJs with erosion had significantly higher VAS pain intensity (36.4 mm vs. 17.3 mm, *p* < 0.001).
Sang et al., 2024 (MRI SR) [44]	Erosion	TMJ arthralgia was associated with condylar erosion, alongside joint effusion, bone marrow edema, and disc displacement.
Jeon et al., 2021 [71]	Condylar flattening	Limitation of mouth opening was associated with flattened/folded disc configuration on MRI.
Jeon et al., 2021 [68]	Subchondral sclerosis	TMJ pain was significantly associated with sclerosis on CBCT (OR 3.81, *p* = 0.001), the only CBCT finding showing a significant clinical correlation in this study.

**Table 9:** Existing TMJ classification systems and their imaging basis.

Classification System	Imaging Modality	Stages/Categories Presented	Osseous Modifications per Stage	Clinical Correlation	Treatment Implication
Wilkes Classification (Stages I–V)[55]	MRI/CBCT/Arthroscopy	Stage I: early; II: early/intermediate; III: intermediate; IV: intermediate/late; V: late	Stage I–II: no/minimal osseous change. Stage III: early osteophyte formation and mild deformity may begin. Stage IV: moderate-to-marked osseous changes—deformity, osteophytes, erosion. Stage V: gross osseous degeneration—severe deformity, erosion, multiple osteophytes, possible bone-to-bone contact.	Stage I–II usually correspond to clicking/disc displacement with reduction; Stage III–IV to intermittent locking and pain; Stage V to chronic pain, crepitus and restricted mouth opening.	Stage I–III: conservative management (splints, physiotherapy, arthrocentesis). Stage IV: minimally invasive surgery (arthroscopy) often considered. Stage V: open joint surgery/reconstruction frequently required.
Emshoff et al., [29]—Subchondral Bone Alteration (SBA) Grading (Grades 0–4)	CBCT	Grade 0; Grade 1; Grade 2; Grade 3; Grade 4	Grade 0: no SBA. Grade 1: single isolated finding (erosion OR osteophyte OR cyst OR sclerosis OR flattening). Grade 2: erosion combined with osteophyte or cyst. Grade 3: resorption with/without one additional SBA finding. Grade 4: resorption plus erosion plus osteophyte or cyst, with/without an additional SBA finding.	Higher grades were more closely associated with unilateral DC/TMD arthralgia in the originating study cohort.	Not explicitly tied to treatment thresholds in the source study; intended primarily as a structured CBCT severity index for research/diagnostic stratification.
Yap et al. [59]—CBCT DJD Staging (NT/ET/LT)	CBCT	NT (no TMJ DJD); ET (early TMJ DJD); LT (late TMJ DJD)	NT: no degenerative findings. ET: defined mainly by loss of cortical continuity/surface erosion. LT: sclerosis, osteophytes, deviation in condylar form, and cyst-like lesions.	Not directly statistically correlated with symptoms in the source study; used primarily as a descriptive radiographic severity grouping.	Not explicitly addressed in the source study.
Hegab MRI-Based TMJ Internal Derangement Classification (Stages 0–4) [47]	MRI	Stage 0: normal; Stage 1A/1B: normal condyle-disc-fossa relationship with effusion/LPM change or degenerative/disc change; Stage 2A–C: anterior disc displacement with reduction (with effusion/LPM, degenerative change, or hypertranslation); Stage 3A–C: anterior disc displacement without reduction (with effusion/LPM, degenerative/stuck-disc change, or pseudodisc/perforation); Stage 4: posterior disc displacement	Degenerative/bone-change subtypes (1B, 2B, 3B) explicitly incorporate condylar/disc degenerative changes as a grading criterion within each broader stage, with severity increasing from stage 1 to stage 3/4.	Designed to integrate disc position, effusion, and degenerative bone change into a single staged framework intended to correlate with clinical TMD presentation.	Authors proposed this staged framework partly to guide management decisions according to derangement severity, though specific treatment thresholds were not detailed in the extracted text.
Piper MRI Diagnostic System (Stages I–Vb) [61]	MRI	Stage I: normal; II: intermittent click; IIIa: lateral pole click; IIIb: lateral pole lock; IVa: medial pole click; IVb: medial pole lock; Va: perforation with acute degenerative joint destruction; Vb: perforation with chronic degenerative joint destruction	Stages I-IV: primarily disc-position/click-lock findings with minimal-to-moderate osseous involvement. Stages Va-Vb: disc perforation accompanied by acute (Va) or chronic (Vb) degenerative joint destruction, i.e., marked osseous breakdown.	Higher stages (IVa/b, Va/b) correspond to more advanced mechanical symptoms (locking) and chronic joint destruction/pain.	Not detailed in the extracted text; advanced stages (V) are conventionally associated with greater likelihood of surgical consideration given joint destruction.
Koyama et al. Condylar Bone-Change Types (N/F/E/D/S) [69]	CBCT	N (normal); F (flattening); E (erosion); D (deformity); S (sclerosis)—applied alongside separate 0–4 erosion severity and 0–3 osteophyte severity scores	Each letter denotes a distinct osseous change type (flattening, erosion, deformity, sclerosis) rather than a sequential severity stage; erosion and osteophyte severity were further graded numerically within this scheme.	Not directly statistically tested for symptom correlation within the extracted text.	Not addressed in the extracted text.
Bianchi et al. [27]—Ordinal Severity Scoring (0–3) for Five Osseous Signs	High-resolution CBCT	0–3 ordinal scale applied separately to flattening, osteophytes, sclerosis, erosion, and cysts	Score 0: absent. Increasing scores (1–3) reflect increasing severity of each individual sign; final diagnostic categories combined these scores into osteoarthritis/DJD, arthralgia, osteoarthrosis/overdiagnosis, or asymptomatic-control groups.	Erosion, osteophytes, and cysts better differentiated symptomatic condylar DJD from controls than flattening or sclerosis, though overdiagnosis was a concern even in asymptomatic individuals.	Not directly addressed; primarily a diagnostic-accuracy/overdiagnosis discussion rather than a treatment framework.
Ahmed et al. [46] CBCT Imaging Criteria (No OA/Indeterminate/OA)	CBCT	No osteoarthritis; Indeterminate; Osteoarthritis (indeterminate often grouped with ‘no OA’ in practice)	‘OA’ category requires definite osseous findings (e.g., erosion, flattening, sclerosis, osteophytes); ‘indeterminate’ reflects subtle/equivocal findings not meeting full diagnostic threshold.	Used as the reference imaging-based diagnostic standard against which clinical DC/TMD groups were compared in several included studies.	Not a treatment-staging system; used purely as a diagnostic imaging classification.
Nayak et al. [72]—CBCT-Based TMJ-Spine Degenerative Severity Index (Grades 0–3)	CBCT	Grade 0: normal; Grade 1: mild; Grade 2: moderate; Grade 3: severe osseous degenerative changes (applied jointly to TMJ and C1-C3 cervical vertebrae)	Grade-specific osseous descriptors were not detailed beyond mild/moderate/severe; the index combines TMJ condylar changes with cervical spine degenerative findings into a single composite severity grade.	Designed to explore a joint TMJ-cervical spine degenerative association, implying that more severe TMJ change may parallel more severe cervical degeneration.	Not addressed in the extracted text.
Dimitroulis Staging (Stages II–IV, used alongside Wilkes) [11]	MRI/Arthroscopy	Stage II-IV reported in the source study (lower/higher stages not specified in extracted text)	Parallels Wilkes staging conceptually, with increasing osseous/structural joint deterioration from Stage II to Stage IV.	Used alongside Wilkes, Vogl MRI criteria, and synovitis/chondromalacia grading to characterize overall joint severity.	Not detailed beyond its role as a comparative staging classification in the source study.

**Table 10:** GRADE-informed certainty assessment of the main findings from the literature review.

Main Finding	Evidence from the Review	Main Limitation	Certainty
CBCT detects recurring TMJ bone changes	Many studies reported flattening, erosion, sclerosis, osteophytes, cortical defects, cystic changes, joint-space changes, and condylar deformation.	Different imaging protocols and terminology.	Moderate
TMJ bone changes may follow an evolution pattern	Studies described changes ranging from early remodeling to advanced degeneration.	Mostly cross-sectional studies; true chronological progression was not proven.	Low
Advanced bone changes are more often linked with symptoms	Several studies reported pain, joint sounds, limited mouth opening, deviation, or dysfunction together with advanced imaging findings.	Few studies directly tested one bone change against one clinical symptom.	Low
Individual bone changes may suggest disease severity	Flattening, sclerosis, erosion, osteophytes, cysts, and deformation were interpreted as different levels of structural involvement.	Prognosis was mostly inferred, with limited longitudinal confirmation.	Low
Treatment approach may vary with severity	Mild findings were usually linked with conservative care; advanced degeneration with multidisciplinary or surgical evaluation.	Treatments were not consistently validated for each specific CBCT finding.	Very low to low
Existing classifications do not provide a dedicated CBCT bone scale	DC/TMD, RDC/TMD, Wilkes, Piper, and MRI-based systems mainly focus on diagnosis, disc position, or internal derangement.	Existing systems were not designed specifically for CBCT osseous staging.	Moderate
Clear definitions can improve reproducibility	Repeated CBCT terms were grouped into common categories such as flattening, erosion, sclerosis, osteophytes, cortical defects, and joint-space changes.	Definitions were harmonized from heterogeneous studies and need external validation.	Low to moderate

**Table 11:** Proposed CBCT-based staging scale for TMJ osseous alterations.

Stage	Main CBCT Criteria	Joint-Space and Dimensional Features	Cortical/Fossa/Eminence Features
Stage I	Preserved condylar morphology and dimensions; centered condyle; normal fossa morphology.	Balanced anterior, superior, and posterior joint spaces; preserved condylar width, anteroposterior diameter, and height.	Intact cortical bone; no erosion, sclerosis, crateriform defects, or advanced remodeling; normal articular eminence inclination.
Stage II	Altered condylar position, commonly anterior and/or medial displacement; early adaptive remodeling.	Reduced anterior joint space with increased posterior joint space; superior space usually preserved or only mildly affected.	Cortical continuity preserved; possible early fossa remodeling, flattening of the articular tubercle, or increased eminence inclination.
Stage III	Reduced condylar dimensions; condylar remodeling; more evident structural alteration.	Marked superior joint-space reduction; possible areas of direct bone contact; reduced condylar height and width.	Cortical thinning; surface remodeling; possible sclerosis, flattening, or limited cortical alteration.
Stage IV	Pronounced condylar deformation; severe structural alteration; end-stage degeneration when ankylosis is present.	Persistent bone-to-bone contact; severe joint-space collapse; marked reduction of condylar height or volume.	Crateriform cortical lesions; extensive cortical disruption; advanced remodeling of articular structures.

**Table 12:** Comparison of Existing CBCT-Based TMJ Classification Systems and the Proposed Four-Stage Framework.

Classification System	Osseous Features	Grading Method	Validation and Reliability	Main Characteristics
Emshoff et al. [29]	Erosion, osteophytes, cysts, sclerosis, flattening, resorption	Grades 0–4; hierarchical combinations of osseous findings	Association with DC/TMD arthralgia reported; interobserver reliability not established from extracted information	Composite subchondral bone severity grading
Yap et al. [59]	Cortical discontinuity, erosion, sclerosis, osteophytes, condylar deformation, cysts	NT, ET, LT; qualitative morphological criteria	Reliability and external validation not established from extracted information	Three-category degenerative joint disease classification
Bianchi et al. [27]	Flattening, osteophytes, sclerosis, erosion, cysts	Ordinal scores 0–3 for five individual findings	Diagnostic associations investigated; staging reliability not established from extracted information	Multisign severity scoring focused on degenerative changes
Nayak et al. [72]	TMJ degeneration and cervical vertebral changes	Grades 0–3; qualitative severity categories	External validation and reliability not established from extracted information	Composite TMJ-cervical degeneration index
Koyama et al. [69]	Flattening, erosion, deformation, sclerosis, osteophytes	Morphological categories N/F/E/D/S; additional erosion and osteophyte severity scores	External validation and reliability not established from extracted information	Classification of individual condylar bone-change patterns

NT = no temporomandibular joint degenerative joint disease; ET = early degenerative joint disease; LT = late degenerative joint disease. Validation and reliability descriptions are based on information extracted in the current manuscript; they should not be interpreted as evidence that the original studies lack such assessments.

## Data Availability

The study protocol was registered on the Open Science Framework (OSF), and the supporting materials and extracted data are publicly available in the repository under registration code z3bt5.

## Supplementary Materials

The following supporting information can be downloaded at: https://www.mdpi.com/article/10.3390/medicina62102031/s1, File S1: Completed PRISMA 2020 checklist [91], which can be downloaded from the online Open Science Framework repository associated with this review, registration code: z3bt5.

## Abbreviations

The following abbreviations are used in this manuscript:

**Abbreviation**	**Definition**
AI	Artificial intelligence
AMSTAR 2	A Measurement Tool to Assess Systematic Reviews 2
AND	Boolean operator AND
BMC	Not explicitly defined in the manuscript
CBCT	Cone-beam computed tomography
CC BY	Creative Commons Attribution
CT	Computed tomography
DC/TMD	Diagnostic Criteria for Temporomandibular Disorders
DJD	Degenerative joint disease
ET	Early TMJ degenerative joint disease
GRADE	Grading of Recommendations Assessment, Development and Evaluation
HNCC	Not explicitly defined in the manuscript
IT	Infrared thermography
JBI	Joanna Briggs Institute
JVA	Not explicitly defined in the manuscript
LPM	Not explicitly defined in the manuscript
LT	Late TMJ degenerative joint disease
MeSH	Medical Subject Headings
MRI	Magnetic resonance imaging
NA	Not applicable
NOS	Newcastle–Ottawa Scale
NT	No TMJ degenerative joint disease
OA	Osteoarthritis
OPG	Not explicitly defined in the manuscript
OR	Boolean operator OR; also used for odds ratio
PDWI	Not explicitly defined in the manuscript
PRISMA	Preferred Reporting Items for Systematic Reviews and Meta-Analyses
RDC/TMD	Research Diagnostic Criteria for Temporomandibular Disorders
SBA	Subchondral bone alteration
SPECT/CT	Single-photon emission computed tomography/computed tomography
SR	Systematic review
TMD	Temporomandibular disorder
TMJ	Temporomandibular joint
USG	Ultrasonography
VAS	Visual analogue scale

## References

[1] Kapos F.P., Exposto F.G., Oyarzo J.F., Durham J. (2020). Temporomandibular Disorders: A Review of Current Concepts in Aetiology, Diagnosis and Management. Oral Surg..

[2] Palmer J., Durham J. (2021). Temporomandibular Disorders. BJA Educ..

[3] Abbass M.M.S., Rady D., El Moshy S., Ahmed Radwan I., Wadan A.-H.S., Dörfer C.E., El-Sayed K.M.F. (2024). The Temporomandibular Joint and the Human Body: A New Perspective on Cross Talk. Dent. J..

[4] Khan J., Singer S.R., Young A., Tanaiutchawoot N., Kalladka M., Mupparapu M. (2023). Pathogenesis and Differential Diagnosis of Temporomandibular Joint Disorders. Dent. Clin. N. Am..

[5] Borges R.E.A., Mendonça L.d.R.A., dos Santos Calderon P. (2024). Diagnostic and Screening Inventories for Temporomandibular Disorders: A Systematic Review. CRANIO^®^.

[6] Klasser G.D., Goulet J.-P., Moreno-Hay I. (2023). Classification and Diagnosis of Temporomandibular Disorders and Temporomandibular Disorder Pain. Dent. Clin. N. Am..

[7] Jalolova R.A., Boymuradov S.A., Djalalova N.A. (2026). Evolution of Diagnostic Strategies for Temporomandibular Joint Disorders: A Scoping Review (2009–2025). J. Maxillofac. Oral Surg..

[8] Azlağ Pekince K., Pekince A., Kazangirler B.Y. (2025). Improving TMJ Diagnosis: A Deep Learning Approach for Detecting Mandibular Condyle Bone Changes. Diagnostics.

[9] Orhan K., Driesen L., Shujaat S., Jacobs R., Chai X. (2021). Development and Validation of a Magnetic Resonance Imaging-Based Machine Learning Model for TMJ Pathologies. BioMed Res. Int..

[10] Maes M., Shrestha S., Keith D.A., Handa S., Lee Y.-H., Van Der Cruyssen F., Verhelst P., Sillmann Y.M., Jacobs R., Guastaldi F.P.S. (2026). Artificial Intelligence in Imaging-Based Diagnosis of Temporomandibular Disorders: A Systematic Review and Meta-Analysis of Diagnostic Accuracy. Digit. Dent. J..

[11] Manek M., Maita I., Bezerra Silva D.F., Pita de Melo D., Major P.W., Jaremko J.L., Almeida F.T. (2025). Temporomandibular Joint Assessment in MRI Images Using Artificial Intelligence Tools: Where Are We Now? A Systematic Review. Dentomaxillofac. Radiol..

[12] Mathew A., Maniangat Luke A., Rastogi S., Singh V. (2026). Is MRI Still the Gold Standard for Assessing Disc Position in Temporomandibular Joint Disorders? A Clinical-Imaging Agreement Study. CRANIO^®^.

[13] Dolwick M.F., Widmer C.G. (2024). Temporomandibular Joint Surgery: The Past, Present, and Future. Int. J. Oral Maxillofac. Surg..

[14] Sathish S., Jain A. (2026). The Sivan Three-Dimensional Cone-Beam Computed Tomography Volumetric Imaging Protocol: A Novel Dual-Mode Rendering Technique for Integrated Visualization of Temporomandibular Joint Disc and Osseous Structures to Evaluate Temporomandibular Joint Disorders. Oral Radiol..

[15] Standerwick R.G., Twilt M., Yen E.H.K., Roberts W.E. (2026). Mechanobiologic Modulation of TMJ Loading in Juvenile Idiopathic Arthritis. CRANIO^®^.

[16] Standerwick R.G., Yen E.H.K., Roberts W.E. (2026). The Temporomandibular Ligament as a Tethered Radius Guiding Articular Eminence Development during Craniofacial Growth. J. Anat..

[17] PRISMA Statement. https://www.prisma-statement.org.

[18] Wang S., Li H., Scells H., Locke D., Zuccon G. (2022). MeSH Term Suggestion for Systematic Review Literature Search. Proceedings of the 25th Australasian Document Computing Symposium.

[19] Hammer P., Rudeanu S.S. (2012). Boolean Methods in Operations Research and Related Areas.

[20] Newcastle-Ottawa Scale (NOS)|Ottawa Hospital Research Institute. https://ohri.ca/en/who-we-are/core-facilities-and-platforms/ottawa-methods-centre/newcastle-ottawa-scale.

[21] De Santis K.K., Pieper D., Lorenz R.C., Wegewitz U., Siemens W., Matthias K. (2023). User Experience of Applying AMSTAR 2 to Appraise Systematic Reviews of Healthcare Interventions: A Commentary. BMC Med. Res. Methodol..

[22] Barker T.H., Stone J.C., Sears K., Klugar M., Leonardi-Bee J., Tufanaru C., Aromataris E., Munn Z. (2023). Revising the JBI Quantitative Critical Appraisal Tools to Improve Their Applicability: An Overview of Methods and the Development Process. JBI Evid. Synth..

[23] Prasad M. (2024). Introduction to the GRADE Tool for Rating Certainty in Evidence and Recommendations. Clin. Epidemiol. Glob. Health.

[24] Santesso N., Glenton C., Dahm P., Garner P., Akl E.A., Alper B., Brignardello-Petersen R., Carrasco-Labra A., de Beer H., Hultcrantz M. (2020). GRADE Guidelines 26: Informative Statements to Communicate the Findings of Systematic Reviews of Interventions. J. Clin. Epidemiol..

[25] OSF. https://osf.io/.

[26] Takaoka R., Koishi Y., Kuyama K., Ueda Y., Ishigaki S., Uchiyama Y., Shimamoto H., Nakatani A., Yatani H. (2022). Cross-Sectional and Longitudinal Assessment of Subchondral Cysts in Temporomandibular Joints: Clinical and MRI Study with a Mean Follow-up of 66 Months. J. Prosthodont. Res..

[27] Bianchi J., Gonçalves J.R., Ruellas A.C.O., Bianchi J.V.P., Ashman L.M., Yatabe M., Benavides E., Soki F.N., Cevidanes L.H.S. (2021). Radiographic Interpretation Using High-Resolution CBCT to Diagnose Degenerative Temporomandibular Joint Disease. PLoS ONE.

[28] Durmuş E.B., Yurdakul F.G., Güler T., Bodur H. (2025). Temporomandibular Joint Disorder in Rheumatoid Arthritis: A Cross-Sectional Ultrasonographic Study. Arch. Rheumatol..

[29] Emshoff R., Bertram A., Hupp L., Rudisch A. (2021). Condylar Erosion Is Predictive of Painful Closed Lock of the Temporomandibular Joint: A Magnetic Resonance Imaging Study. Head Face Med..

[30] Emshoff R., Bertram A., Hupp L., Rudisch A. (2021). A Logistic Analysis Prediction Model of TMJ Condylar Erosion in Patients with TMJ Arthralgia. BMC Oral Health.

[31] Eisazadeh M., Khojastepour L., Haghnegahdar A., Soltani P. (2021). Comparison of Condylar Bone Density in Cone-Beam Computed Tomographic Images of Patients with and without Temporomandibular Joint Disorders. J. Orofac. Sci..

[32] Biplob S.J. (2025). Assessment of Bony Changes of Condyle in Patient with Temporomandibular Joint Disorder Diagnosed in a Tertiary Care Hospital. Planet.

[33] Chen J., Yin W., Ding S., Cao J., Ding X., Liang Y., Xiao J. (2026). Application of Fully Digital Occlusal Splints in Painful Temporomandibular Disorders: A Randomized Controlled Clinical Study. Head Face Med..

[34] Rady N.A., Bahgat M.M., Abdel-Hamid A.M. (2022). Promising Minimally Invasive Treatment Modalities for Symptomatic Temporomandibular Joint Disc Displacement with Reduction: A Randomized Controlled Clinical Trial. BMC Oral Health.

[35] Ritto F.G., Cueto A.P., Dos Santos Canellas J.V., Zuniga J.R., Tiwana P.S., Pimentel T., Medeiros P.J. (2022). Arthrocentesis versus Nonsurgical Methods in the Management of Temporomandibular Joint Closed Lock and Pain: A Double-Blind Randomized Controlled Trial. Oral Surg. Oral Med. Oral Pathol. Oral Radiol..

[36] Jia X.-Y., Jing S.-L., Sun Y., Gong Z.-C., Guo Z.-C. (2024). A Randomized Controlled Clinical Trial of Concentrated Growth Factor Combined with Sodium Hyaluronate in the Treatment of Temporomandibular Joint Osteoarthritis. BMC Oral Health.

[37] Zheng X., Guo L., Yang S. (2026). Concentrated Growth Factor as Treatment for Temporomandibular Joint Osteoarthritis: A Randomized Controlled Clinical Trial. J. Cranio-Maxillofac. Surg..

[38] Sharma J., Banerjee R., Gajbhiye V., Bhoge A., Jaiswal P., Nayak K. (2025). Randomized Clinical Trial to Evaluate the Effectiveness of Different Thicknesses of Stabilization Splints in Treating Temporomandibular Disorder Using Joint Vibration Analysis as a Novel Diagnostic Tool. J. Indian Prosthodont. Soc..

[39] Cömert Kılıç S. (2021). Does Glucosamine, Chondroitin Sulfate, and Methylsulfonylmethane Supplementation Improve the Outcome of Temporomandibular Joint Osteoarthritis Management with Arthrocentesis plus Intraarticular Hyaluronic Acid Injection. A Randomized Clinical Trial. J. Cranio-Maxillofac. Surg..

[40] Li H., Han W., Wu X., Feng J., Li G., Meng J. (2024). Preliminarily study of arthrocentesis combined with liquid phase concentrated growth factor injection in the treatment of unilateral temporomandibular joint osteoarthritis. Beijing Da Xue Xue Bao.

[41] Ren L., Chen P., Musa M., Zhao Y., Awad R., Xiao Z., Li C., Li D., Chen X. (2025). Quantitative and Qualitative Condylar Changes Post-Stabilization Splint in Patients with Temporomandibular Disorder and Chewing Side Preference. Sci. Rep..

[42] Musa M., Zhang Q., Awad R., Wang W., Ahmed M.M.S., Zhao Y., Almashraqi A.A., Chen X., Alhammadi M.S. (2023). Quantitative and Qualitative Condylar Changes Following Stabilization Splint Therapy in Patients with Temporomandibular Joint Disorders. Clin. Oral Investig..

[43] Tăut M., Barbur I., Hedeșiu M., Ban A., Leucuța D., Negucioiu M., Buduru S.D., Ilea A. (2024). Condylar Remodeling and Skeletal Changes Following Occlusal Splint and Manual Therapy: A Cone Beam Computed Tomography Study in Temporomandibular Disorders. J. Clin. Med..

[44] Sang S., Ameli N., Almeida F.T., Friesen R. (2024). Association between Clinical Symptoms and MRI Image Findings in Symptomatic Temporomandibular Joint (TMJ) Disease: A Systematic Review. J. Cranio-Maxillofac. Surg..

[45] Januzzi E., Pauletto P., Cunha T.C.A., Silva G., Iwassake M.K., de Pedro M., Fernandez J., Pedrosa A.R., de Souza B.D.M., Duarte A.S.B. (2025). Outcomes of Modified Arthrocentesis Using Concentric Needle and Cannula Technique with Sequential Viscosupplementation and Orthobiologics in Both TMJ Compartments. Sci. Rep..

[46] Ahmed M.M.S., Shi D., Al-Somairi M.A.A., Alhashimi N., Almashraqi A.A., Musa M., Li N., Chen X., Alhammadi M.S. (2023). Three Dimensional Evaluation of the Skeletal and Temporomandibular Joint Changes Following Stabilization Splint Therapy in Patients with Temporomandibular Joint Disorders and Mandibular Deviation: A Retrospective Study. BMC Oral Health.

[47] Hegab A.F., Al Hameed H.I., Karam K.S. (2021). Classification of Temporomandibular Joint Internal Derangement Based on Magnetic Resonance Imaging and Clinical Findings of 435 Patients Contributing to a Nonsurgical Treatment Protocol. Sci. Rep..

[48] Knezevic M.J., Knezevic A., Boban J., Maletin A., Milekic B., Koprivica D.D., Puskar T., Semnic R. (2023). High-Field Magnetic Resonance Imaging of the Temporomandibular Joint Low Agreement with Clinical Diagnosis in Asymptomatic Females. Diagnostics.

[49] de Lima E.D., Souza Paulino J.A., Lira de Farias Freitas A.P., Viana Ferreira J.E., Barbosa J.d.S., Bezerra Silva D.F., Bento P.M., Araújo Maia Amorim A.M., Melo D.P. (2022). Artificial Intelligence and Infrared Thermography as Auxiliary Tools in the Diagnosis of Temporomandibular Disorder. Dento Maxillo Facial Radiol..

[50] González L.V., López J.P., Díaz-Báez D., Gómez-Delgado A. (2021). Correlation between MRI-Diagnosed Joint Effusion and Demographic, Clinical, Imaging, and Arthroscopic Findings of the Temporomandibular Joint. J. Cranio-Maxillofac. Surg..

[51] Mao W.-Y., Fang Y.-Y., Wang Z.-Z., Liu M.-Q., Sun Y., Wu H.-X., Lei J., Fu K.-Y. (2025). Automated Diagnosis and Classification of Temporomandibular Joint Degenerative Disease via Artificial Intelligence Using CBCT Imaging. J. Dent..

[52] Dumanlı D., Şeker Ç. (2025). Ultrasonographic and Radiographic Evaluation of Osteoarthritic Changes in the Temporomandibular Joint. Diagnostics.

[53] Wu M., Lai H., Almeida F.T., Friesen R. (2025). Exploring the Association Between Clinical Features and CBCT Findings in TMJ Degenerative Joint Disease. J. Oral Rehabil..

[54] Hoa N.N., Do T., Binh T.T., Dong T.T., Quyen T.T.M., Hoang V.H. (2025). Evaluation of Mandibular Functional Limitation in Patients with Temporomandibular Joint Disorders and the Relationship with Other Psychosocial Factors. PLoS ONE.

[55] Bertotti M., de la Sen Ó., Bascones A.E., Amorós C.M., Mérida-Velasco J.R., Martín-Granizo R., de Moraes L.O.C. (2025). Correlation of Temporomandibular Joint Effusion on MRI with Wilkes Staging, Arthroscopic Findings, and Synovial Fluid Biomarkers in Temporomandibular Disorders. J. Cranio-Maxillofac. Surg..

[56] Topaloglu Yasan G., Adiloglu S., Tuz H.H., Sahar D. (2023). Evaluation of Clinical Signs and Magnetic Resonance Imaging Findings in Patients with Temporomandibular Disorders. J. Cranio-Maxillofac. Surg..

[57] Mizuhashi F., Ogura I., Mizuhashi R., Watarai Y., Oohashi M., Suzuki T., Saegusa H. (2023). Examination for the Factors Involving to Joint Effusion in Patients with Temporomandibular Disorders Using Magnetic Resonance Imaging. J. Imaging.

[58] Fernández-Ferro M., Fernández-González V., Salgado-Barreira Á., Santos-Armentia E., Valdés-Sarmiento P., Fernández-García A., Gómez-Rey D., Fernández-Sanromán J. (2023). Correlation between the Main Clinical, Imaging, and Arthroscopy Findings in Patients with Temporomandibular Disorders. Int. J. Oral Maxillofac. Surg..

[59] Yap A.U., Lei J., Zhang X.-H., Fu K.-Y. (2023). TMJ Degenerative Joint Disease: Relationships between CBCT Findings, Clinical Symptoms, and Signs. Acta Odontol. Scand..

[60] Collin M., Hagelberg S., Ernberg M., Hedenberg-Magnusson B., Christidis N. (2022). Temporomandibular Joint Involvement in Children with Juvenile Idiopathic Arthritis-Symptoms, Clinical Signs and Radiographic Findings. J. Oral Rehabil..

[61] Tegnander T., Chladek G., Hovland A., Żmudzki J., Wojtek P. (2021). Relationship between Clinical Symptoms and Magnetic Resonance Imaging in Temporomandibular Disorder (TMD) Patients Utilizing the Piper MRI Diagnostic System. J. Clin. Med..

[62] Schnabl D., Pamminger D., Rohde S., Hupp L., Rudisch A., Emshoff R. (2025). Prevalence of Degenerative Alterations of Osseous Temporomandibular Joint Structures in Cone Beam Computed Tomograms in a Consecutive Sample of Young Adults. A Retrosp. Study Clin. Oral Investig..

[63] Koshal N., Patil D.J., Laller S., Malik M., Punia R.S., Sawhney H. (2021). Assessment of Correlation Between Bone Quality and Degenerative Bone Changes in Temporomandibular Joint by Computed Tomography—A Retrospective Study. J. Indian Acad. Oral Med. Radiol..

[64] Nina-Aguilar L., Ramos-Gómez F., Sánchez-Tito M. (2025). Degenerative Changes of the Mandibular Condyle and Their Relationship with Joint Space: A CBCT Study. J. Clin. Exp. Dent..

[65] Yulek H., Keser G., Pekiner F.N., Unal S.Y. (2026). Evaluation of TMJ Bone Degeneration Using CBCT in Kennedy Class I and II Patients. J. Coll. Physicians Surg. Pak. JCPSP.

[66] Paknahad M., Khojastepour L., Tabatabaei S., Mahjoori-Ghasrodashti M. (2023). Association between Condylar Bone Changes and Eichner Index in Patients with Temporomandibular Dysfunction: A Cone Beam Computed Tomography Study. J. Dent..

[67] Chikkanna M., Parveen S., Swathy S., Punnoose K., Sachidanandan M., Almutairi F.J., Babu J.S., Swarnalatha C., Nayyar A.S. (2026). Morphological Assessment of Temporomandibular Joint Spaces, Mandibular Condyle and Glenoid Fossa in Patients with Generalized Osteo- and Rheumatoid Arthritis: A Clinical and Tomographic Study. Indian J. Otol..

[68] Jeon K.J., Lee C., Choi Y.J., Han S.-S. (2021). Analysis of Three-Dimensional Imaging Findings and Clinical Symptoms in Patients with Temporomandibular Joint Disorders. Quant. Imaging Med. Surg..

[69] Yildizer E., Odabaşı O. (2023). Differences in Clinical and Radiographic Features between Bilateral and Unilateral Adult Degenerative Temporomandibular Joint Disease: A Retrospective Cross-Sectional Study. Int. Orthod..

[70] Görürgöz C., İçen M., Kurt M.H., Aksoy S., Bakırarar B., Rozylo-Kalinowska I., Orhan K. (2023). Degenerative Changes of the Mandibular Condyle in Relation to the Temporomandibular Joint Space, Gender and Age: A Multicenter CBCT Study. Dent. Med. Probl..

[71] Jeon S.-H., Lim S.-W., Jung K.-H., Jeon J.-Y., Kim S.-Y., Kim J.-Y., Choi Y.-Y., Hwang K.-G. (2023). The Clinical Effectiveness of Fused Image of Single-Photon Emission CT and Facial CT for the Evaluation of Degenerative Change of Mandibular Condylar Head. Maxillofac. Plast. Reconstr. Surg..

[72] Nayak A.G., Bhatnagar S., Pai Khot A.J. (2024). Correlation Between Radiological Changes of the Temporomandibular Joint and Upper Cervical Vertebrae in Degenerative Joint Disease: A Cone-Beam Computed Tomography-Based Analytical Study. Cureus.

[73] Ming X., Cheng X., Tian C., Li W., Wang R., Qian C., Zeng X. (2023). Evaluation of Condylar Osseous Changes Using a Wireless Detector with Proton Density-Weighted Imaging Sequences. Quant. Imaging Med. Surg..

[74] Hilgenberg-Sydney P.B., Zanlorenzi F.F., Cunha C.O. (2024). Comparative Analysis of Temporomandibular Joint Morphology in Degenerative Joint Disease: A Cone-Beam CT Study in Patients with and without Arthralgia. Saudi Dent. J..

[75] Sang J., Yang C., Liu H., Zhang M., Xiao Y., Li J., Li G. (2024). Therapeutic Efficacy of Intra-Articular Betamethasone Injection and Comparative Assessment of Temporomandibular Joint Pathologies Using Cone-Beam Computed Tomography and Magnetic Resonance Imaging. Trop. J. Pharm. Res..

[76] Ramesh E., Ganesan A., Lakshmi K.C. (2025). Evaluation of Temporomandibular Joint Disc Elasticity as a Diagnostic Criterion in Temporomandibular Joint Disorders Using Magnetic Resonance Imaging. J. Head Neck Physicians Surg..

[77] Millón Cruz A., Martin-Granizo R., Barone S., Pérez Rodríguez L.M., Menéndez S.M., Falahat Noushzady F., Arias Díaz J. (2024). Reliability of Magnetic Resonance for Temporomandibular Joint Disc Perforation: A 12 Years Retrospective Study. J. Cranio-Maxillofac. Surg..

[78] Wojciechowska B., Szarmach A., Michcik A., Sikora M., Drogoszewska B. (2024). Is Ultrasonography an Effective Method for Diagnosing Degenerative Changes in the Temporomandibular Joint?. Biomedicines.

[79] Farronato M., Crispino R., Fabbro M.D., Tartaglia G.M., Cenzato N. (2025). TMJ Pericapsular Guided Injection with Visual-Inertial Odometry (Augmented/Mixed Reality): A Novel Pilot Clinical Approach for Joint Osteoarthrosis Drug Delivery. J. Dent..

[80] Yeladandi M., Chavva S., Padala S.B., Khanam S., Vemula H., Moparthi H. (2024). Effects of Transcutaneous Electrical Nerve Stimulation, Laser Therapy, and Ultrasound in Managing Temporomandibular Disorders: A Randomised Clinical Study. J. Clin. Diagn. Res..

[81] Mehta V., Vathani A., Kamma P.K.G., Chaturvedi S., Mathur A., Noor T. (2026). Comparing Traditional Versus AI-Assisted TMJ Disorder Management Approaches: A Systematic Review and Meta-Analysis. Clin. Exp. Dent. Res..

[82] Ronsivalle V., Marrapodi M.M., Tirupathi S., Afnan L., Cicciù M., Minervini G. (2024). Prevalence of Temporomandibular Disorders in Juvenile Idiopathic Arthritis Evaluated with Diagnostic Criteria for Temporomandibular Disorders: A Systematic Review with Meta-Analysis. J. Oral Rehabil..

[83] Wu M., Almeida F.T., Friesen R. (2021). A Systematic Review on the Association Between Clinical Symptoms and CBCT Findings in Symptomatic TMJ Degenerative Joint Disease. J. Oral Facial Pain Headache.

[84] Farook T.H., Dudley J. (2023). Automation and Deep (Machine) Learning in Temporomandibular Joint Disorder Radiomics: A Systematic Review. J. Oral Rehabil..

[85] Ehrmann E., Bernabeu M., Tillier Y., Camia J., Ecalle C., Savoldelli C., Charavet C. (2024). Impact of Orthodontic-Surgical Treatments on the Signs and Symptoms of Temporomandibular Disorders: A Systematic Review. Dent. J..

[86] Alvear Miquilena A., Velepucha Torres R., Chauca-Bajaña L., Carrera Trejo M., Loayza Lara S., Pérez-Jardón A., Suarez-Palacios J., Ron B.V. (2024). Prevalence of Reported Temporomandibular Disorders in Children and Adolescents: Systematic Review and Meta-Analysis. Appl. Sci..

[87] Minervini G., Franco R., Marrapodi M.M., Crimi S., Badnjević A., Cervino G., Bianchi A., Cicciù M. (2023). Correlation between Temporomandibular Disorders (TMD) and Posture Evaluated Trough the Diagnostic Criteria for Temporomandibular Disorders (DC/TMD): A Systematic Review with Meta-Analysis. J. Clin. Med..

[88] Dygas S., Szarmach I., Radej I. (2022). Assessment of the Morphology and Degenerative Changes in the Temporomandibular Joint Using CBCT According to the Orthodontic Approach: A Scoping Review. BioMed Res. Int..

[89] Emshoff R., Rudisch A., Bertram S. (2025). Clinical Factors Associated with Radiographic Severity of Progressive Temporo-mandibular Joint Osteoarthritis: A Retrospective CBCT Study. BMC Musculoskelet. Disord..

[90] Semerci Z.M., Günen Yılmaz S. (2024). Evaluation of Rheumatic Diseases Affecting the Temporomandibular Joint: A Cone Beam Computed Tomography Study and Literature Review. Diagnostics.

[91] Page M.J., McKenzie J.E., Bossuyt P.M., Boutron I., Hoffmann T.C., Mulrow C.D., Shamseer L., Tetzlaff J.M., Akl E.A., Brennan S.E. (2021). The PRISMA 2020 statement: An updated guideline for reporting systematic reviews. BMJ.

